# Supplementary material for: Non-viral ex vivo genome-editing in mouse bona fide hematopoietic stem cells with CRISPR/Cas9
Source: Mol Ther Methods Clin Dev. 2021 Jan 9;20:451–62. doi: 10.1016/j.omtm.2021.01.001 (PMC7873578; doi:10.1016/j.omtm.2021.01.001)
Supplement: Document S1. Figures S1–S9 and Tables S6 and S7 [file mmc1.pdf]

OMTM, Volume *20*

Supplemental Information

Non-viral *ex vivo* genome-editing in mouse bona fide hematopoietic stem cells with CRISPR/Cas9

Suvd Byambaa, Hideki Uosaki, Tsukasa Ohmori, Hiromasa Hara, Hitoshi Endo, Osamu Nureki, and Yutaka Hanazono

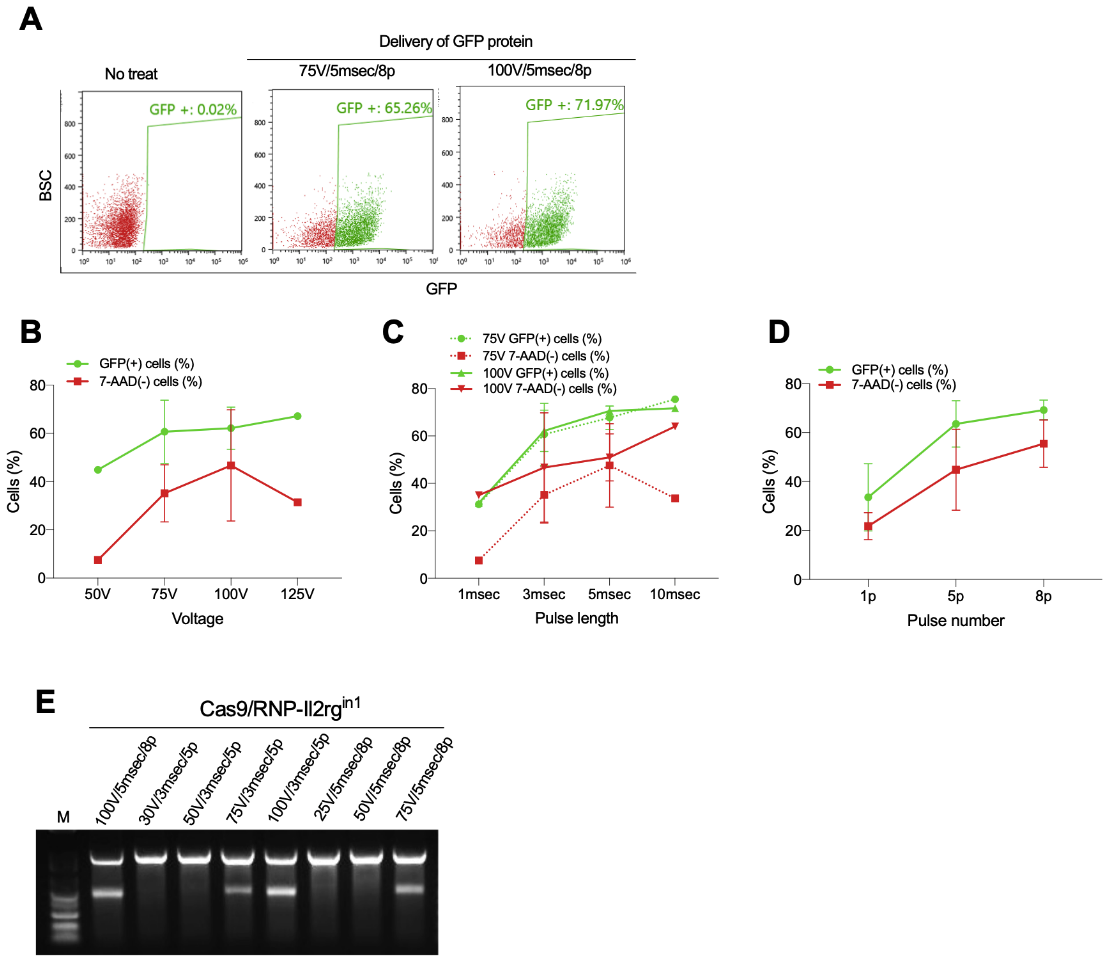

Figure S1. The electroporation conditions were optimized by delivery with GFP protein.

A. Delivery of GFP as a protein to Lin^- cells were assessed by flow cytometry. The conditions of 75 V or 100 V, 5 msec, and 8 pulses for the poring pulse; and those of 10 V, 50 ms, and 5 pulses for the transfer pulse were optimal.

B - D. Optimization of electroporation conditions in Lin^- cells with GFP protein using NEPA21 Super Electroporator. The efficiency of GFP delivery (green) and cell viability (red) were analyzed by flow cytometry. We compared poring pulse parameters: voltage (B), 50 to 125 V with 5 msec and 8 pulses; pulse duration (C), 1 to 10 msec with 100 V and 8 pulses; and pulse number (D), 1 to 8 pulses with 100 V and 5 msec.

E. Surveyor assays at *Il2rg* intron 1 in Lin^- cells at 72 hours after different electroporation conditions to examine DSB at the target site.

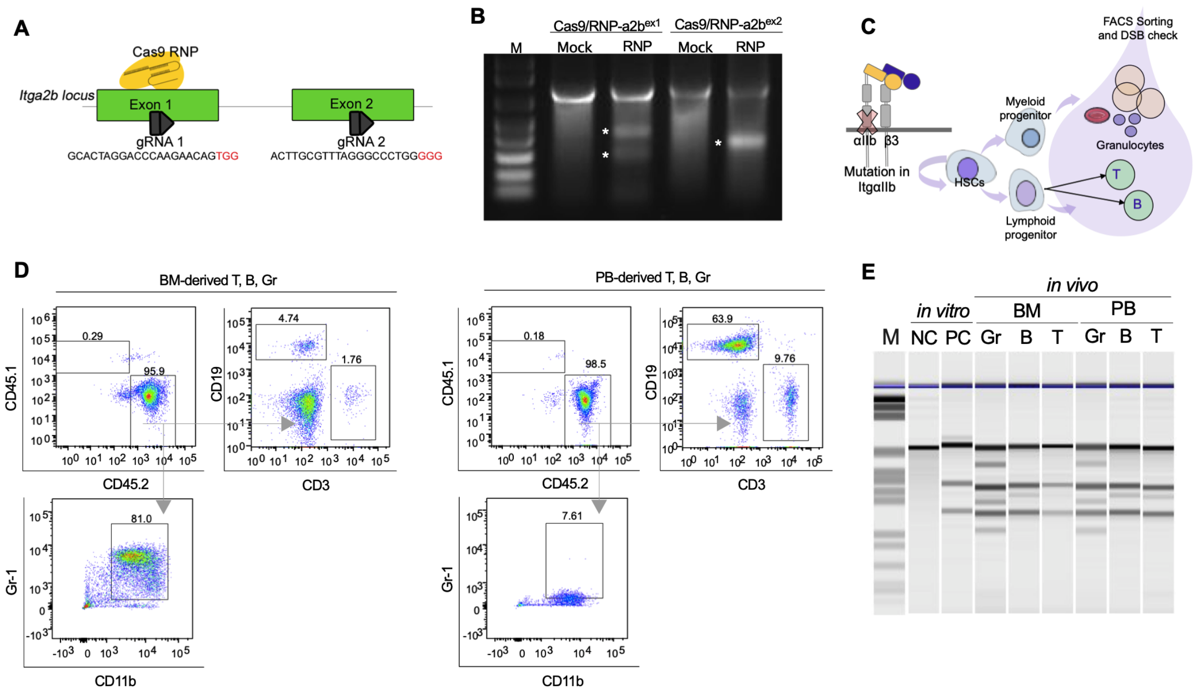

Figure S2. In vitro genome-editing of *Itga2b* in Lin⁻ cells and in vivo detection of the knockout of *Itga2b* from flow cytometry sorted peripheral blood and bone marrow-derived granulocytes and T and B lymphocytes from tertiary recipient.

A. A schematic diagram of the target site in the exon 1 and 2 of the mouse *Itga2b* gene locus.

B. We introduced Cas9/RNP-a2b^{ex1} and Cas9/RNP-a2b^{ex2} to mouse bone marrow Lin^{-} cells by electroporation, and the mutations were examined by Surveyor assay after 5-day culture in vitro.

C. A schematic diagram of knockout of *Itga2b* gene in mouse HSC and flow cytometric sorting of HSC-derived granulocytes and T and B lymphocytes from the bone marrow and peripheral blood of recipients.

D. The detection of mutations in exon 1 of *Itga2b* in sorted granulocytes and T and B lymphocytes from a tertiary recipient after 12 weeks post-transplantation. BM, bone marrow; PB, peripheral blood; Gr, granulocytes; B, B lymphocytes; and T, T lymphocytes; NC, negative control; PC, positive control. PC was the same sample used in Fig 2b that had a site-specific cleavage in vitro.

E. The detection of mutations in exon 1 of *Itga2b* in sorted granulocytes and T and B lymphocytes from a tertiary recipient after 12 weeks post-transplantation.

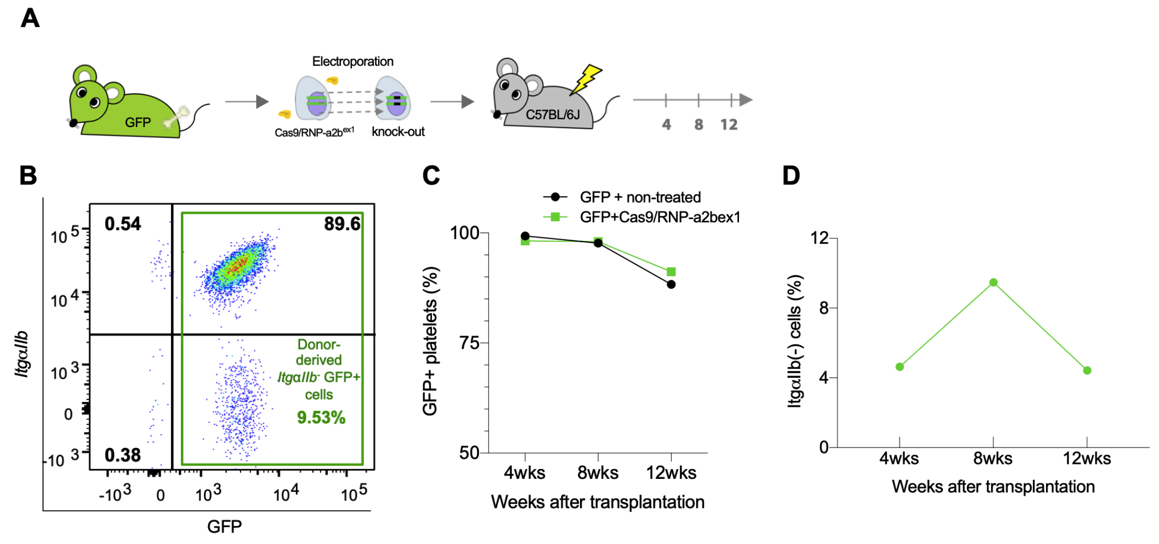

Figure S3. Genome-editing of *Itga2b* using GFP$^+$ Lin$^-$ cells.

A. A schematic diagram of transplantation of GFP$^+$ Lin$^-$ cells after electroporation with Cas9/RNP-a2b^{ex1}.

B. Flow cytometric results of donor-derived (GFP$^+$) ItgαIIb$^-$ platelets at 8 weeks post-transplantation.

C. More than 90% of platelets were positive for GFP; that is, donor-derived.

D. Around 10% of GFP$^+$ platelets were ItgαIIb$^-$; that is, successfully genome-edited donor HSC-derived.

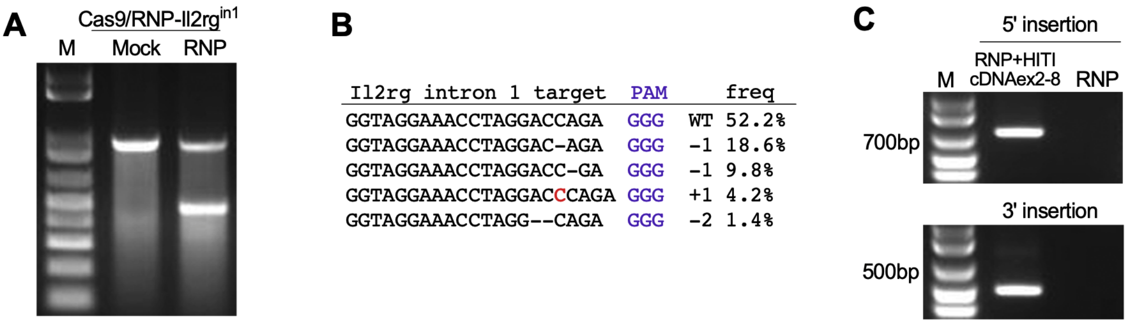

Figure S4. Integration of HITI-cDNAex2-8 into *Il2rg* intron 1 in NIH3T3 cells in vitro.

A. Surveyor assays at *Il2rg* intron 1 in NIH3T3 at 72 hours after electroporation with Cas9/RNP-Il2rg^{in1}.

B. Targeted deep sequencing showing a rate of 43.1% insertions and deletions (InDels) in the cellular genomes after electroporation with Cas9/RNP-il2rg^{in1} (PAM, purple; mutations, red).

C. Genomic PCR results indicating successful knock-in of HITI-cDNA ex2-8 into intron 1 in NIH3T3 cells. The 5'-end insertion was confirmed with the P1+P2 primer set and the 3'-end insertion was confirmed with the P3+P4 primer set.

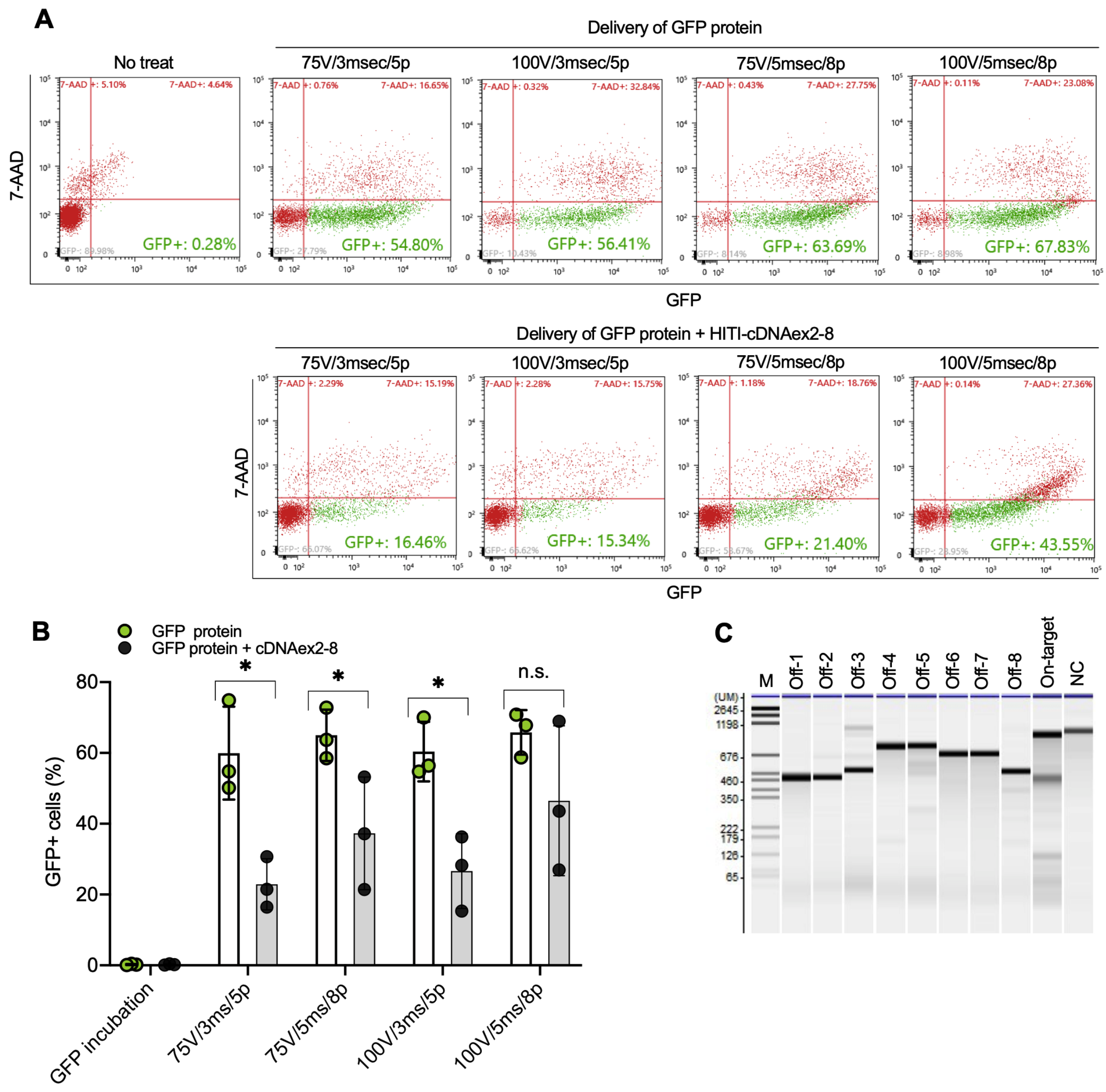

Figure S5. A. Representative plots of flow cytometry with GFP and 7-AAD. Green dots indicate GFP-positive live cells (figures in the plot indicate GFP%).

B. The comparison of electroporation with GFP protein into Lin$^-$ cells by NEPA21 Super Electroporator. The delivery of GFP protein alone (green dots) versus GFP protein with HITI-cDNA ex2-8 plasmid (black dots) was analyzed by flow cytometry. The addition of cDNA significantly reduces the efficiency of protein delivery. Mann-Whitney one-tailed U test: $*p < 0.05$.

C. Assessment of off-target InDels. Putative off-target sites for Cas9/RNP-il2rg^{in1} were analyzed by Surveyor assay in RNP+HITI-cDNA ex2-8 treated Lin$^-$ cells. No specific mutations were observed. Off, putative off-targets; On-target, *Il2rg* Intron 1; NC, negative control.

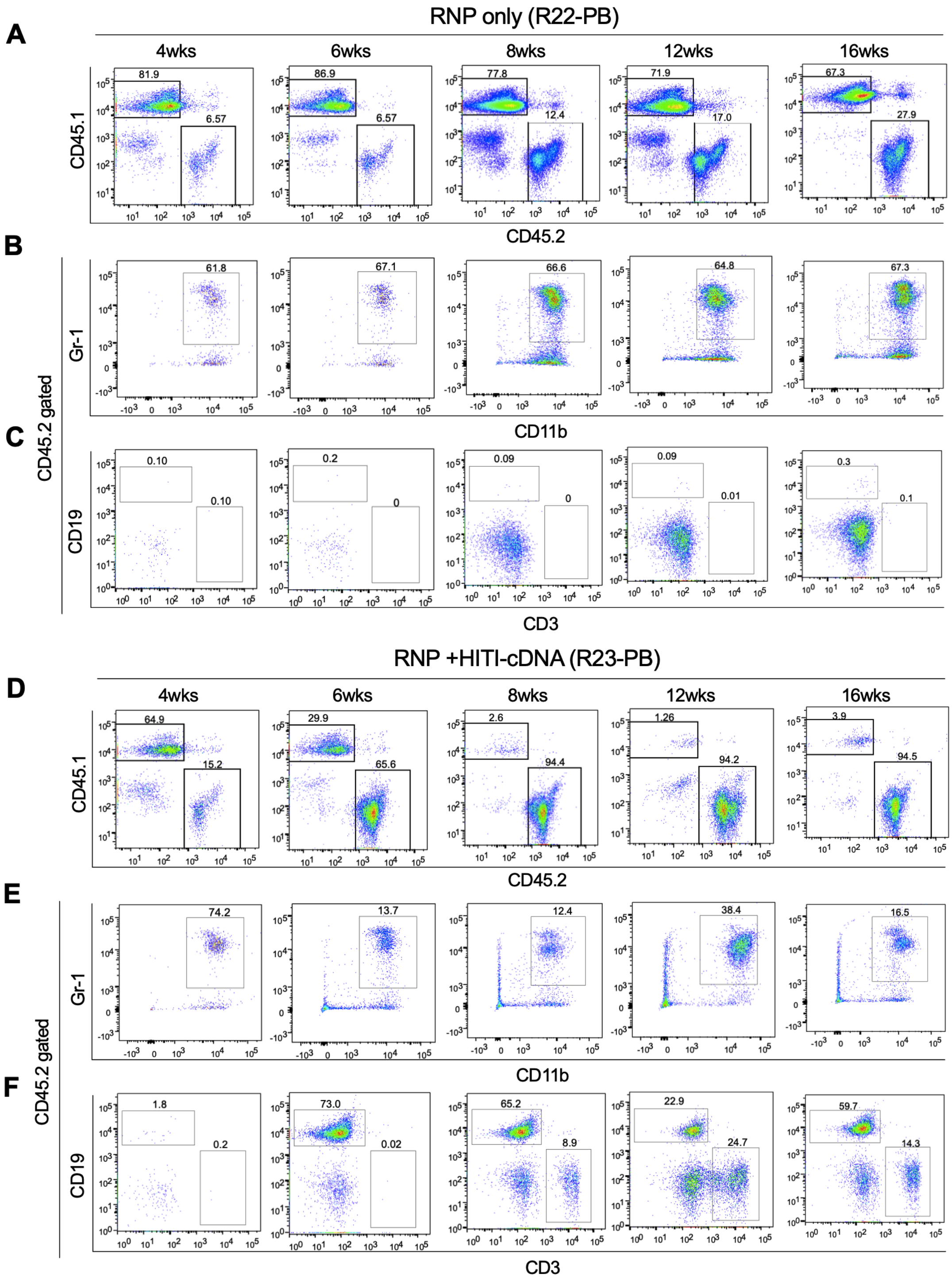

Figure S6. Time course study of donor-derived cells in the primary recipients.

A-C. Plots of CD45.1 (host) and CD45.2 (donor) (**A**), CD45.2-gated Gr-1+ and Cd11b+ granulocytes (**B**), and CD45.2-gated CD19+ B cells and CD3+ T cells (**C**) of a recipient with Lin- cells treated with RNP only.

D-E. Plots of CD45.1/CD45.2 (**D**), CD45.2-gated Gr-1+ and Cd11b+ granulocytes (**E**), and CD45.2-gated CD19+ B cells and CD3+ T cells (**F**) of a recipient with Lin- cells treated with RNP+HITI-cDNA.

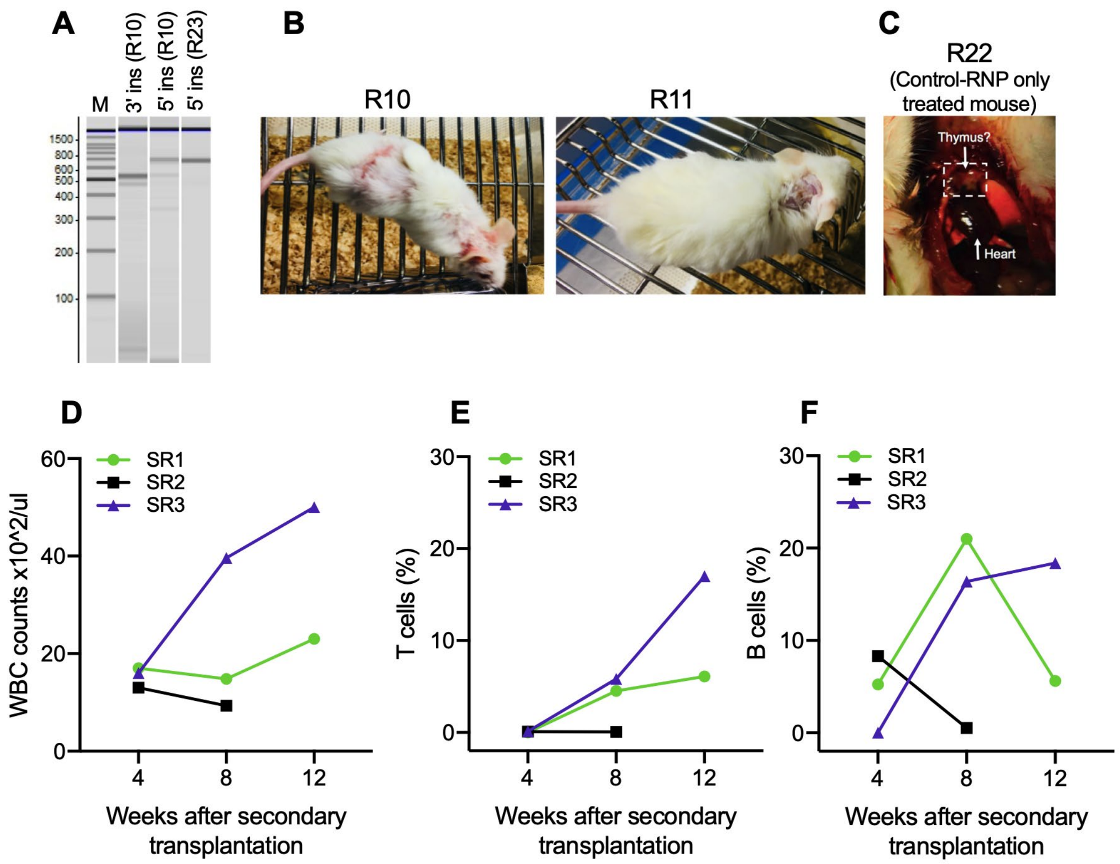

Figure S7. Phenotypical correction of *Il2rg*-mutated mice after transplantation of genome-edited HSCs. In primary transplantation, Lin⁻ cells of *Il2rg*-mutated mice (CD45.2) were transplanted to NOG mice (CD45.1).

A. Genomic PCR confirming the integration of HITI-cDNAex2-8 into intron 1 in primary recipients. The 3'-end insertion was confirmed in primary recipients R10 and R23, however, 5'-end insertion was confirmed only in R10.

B. In the transplantation of Lin⁻ cells of *Il2rg*-mutated mice (CD45.2) to NOG mice (CD45.1) which was allogeneic, graft-versus-host disease (GVHD)-like symptoms such as skin thickening, damage, and rashes appeared after 12 weeks post-transplantation in the primary recipients R10 and R11, suggesting that the reconstituted T lymphocytes were functional.

C. In the control mice (*e.g.* R22) that had received Lin- cells electroporated with the Cas9/RNP-il2rg^{in1}, the development of thymuses was not observed at 20 weeks after transplantation.

D – F. Secondary recipients (SR1-3) of *Il2rg*-mutated mice were transplanted with Lin⁻ cells from the cured mice after primary transplantation. White blood cell counts **(D)**, and fractions of CD3$^+$ T cells **(E)** and CD19$^+$ B cells **(F)** in the secondary recipients at 4 to 12 weeks after transplantation.

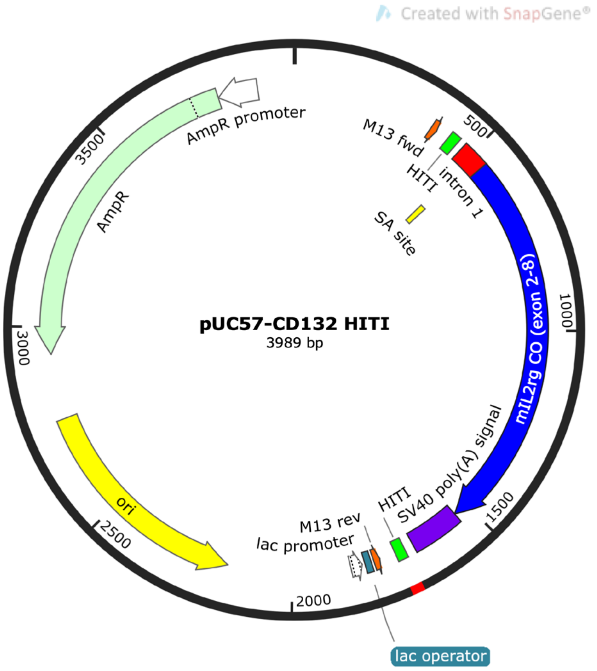

Figure S8. Plasmid map of HITI-cDNAex2-8 (pUC57-CD132-HITI). A plasmid of 3989 base pairs designed for cDNA of *Il2rg* exon 2 to 8. *mIl2rg* CO (exon2-8), codon optimized cDNA of exon 2 to 8 sequence; HITI, Homology independent targeted integration site; AmpR, ampicillin resistance; ori, origin of replication; and SV40 poly A, polyadenylation signal.

M13 forward
GTAAAACGACGGCCAGTGAATTCGAGCTCGGTACCTCGCGAATGCATCTAG
HITI
ACTCCCTCTGGTCCTAGGTTTCCTACCCTAGATAGATCTGTCGA
cDNA_intron1
CCACTGCTGCTTCTTCTGACCAAGAATTCTTTTTCTTTCACTCCACTATTTCATTTTCTTCCCAAACTTAG
cDNA_exon2-8
ATCTGATCCTGACCAGCACCGCGCCGGAACACCTGAGCGCGCCGACCCTGCCGCTGCCGGAGGTTCAATGCTT
CGTTTTCAACATCGAGTATATGAACTGCACCTGGAACAGCAGCAGCGAGCCGCAGGCGACCAACCTGACCCT
GCACTACCGTTATAAGGTGAGCGACAACAACACCTTCCAAGAGTGCAGCCACTACCTGTTTAGCAAGGAAATC
ACCAGCGGTTGCCAGATCCAAAAAGAAGACATTCAGCTGTATCAAACCTTCGTGGTTCAGCTGCAAGATCCGC
AGAAGCCGCAACGTCGTGCGGTGCAGAAACTGAACCTGCAAAACCTGGTTATTCCGCGTGCGCCGGAGAACC
TGACCCTGAGCAACCTGAGCGAGAGCCAGCTGGAACTGCGTTGGAAGAGCCGTCACATCAAAGAGCGTTGCC
TGCAGTACCTGGTGCAATATCGTAGCAACCGTGACCGTAGCTGGACCGAGCTGATTGTTAACCACGAACCGCG
TTTCAGCCTGCCGAGCGTGGATGAGCTGAAGCGTTACACCTTTCGTGTTCGTAGCCGTTATAACCCGATCTGCG
GTAGCAGCCAGCAATGGAGCAAATGGAGCCAGCCGGTGCACTGGGGCAGCCACACCGTTGAGGAAAACCCG
AGCCTGTTCGCGCTGGAGGCGGTGCTGATCCCGGTTGGTACCATGGGCCTGATCATTACCCTGATTTTCGTGTA
CTGCTGGCTGGAACGTATGCCGCCGATCCCGCCGATTAAGAACCTGGAGGACCTGGTGACCGAATATCAGGG
CAACTTCAGCGCGTGGAGCGGTGTTAGCAAAGGCCTGACCGAGAGCCTGCAACCGGATTACAGCGAGCGTTT
TTGCCACGTTAGCGAAATTCCGCCGAAAGGTGGCGCGCTGGGTGAAGGTCCGGGTGGTAGCCCGTGCAGCCT
GCATAGCCCGTACTGGCCGCCGCCGTGCTACAGCCTGAAGCCGGAAGCGTAA
cDNA_polyA
AACTTGTTTATTGCAGCTTATAATGGTTACAAATAAAGCAATAGCATCACAAATTTCACAAATAAAGCATTTTT
TTCACTGCATTCTAGTTGTGGTTTGTCCAAACTCATCAATGTATCTTATCATGTCTGGATCCTCGAGCGGCCGC
ATCGGATCCCG
HITI
ACTCCCTCTGGTCCTAGGTTTCCTACCCGTCGACTGCAGAGGCCTGCATGCAAGCTTGGCGTAATCATG
M13 reverse
GTCATAGCTGTTTCCTG

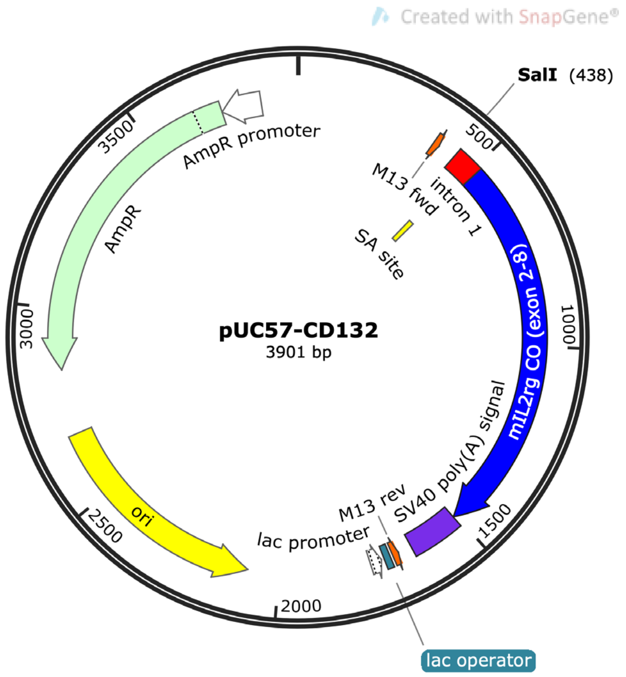

Figure S9. Plasmid map RE-cDNAex2-8 (pUC57-CD132). A plasmid of 3901 base pairs designed for cDNA of *Il2rg* exon 2 to 8. *mIl2rg* CO (exon2-8), codon optimized cDNA of exon 2 to 8 sequence; AmpR, ampicillin resistance; ori, origin of replication; SV40 poly A, polyadenylation signal; and SalI, restriction enzyme site was used for linearization.

M13 forward
GTAAAACGACGGCCAGT GAATTCGAGCTCGGTACCTCGCGAATGCATCTAGATAGATCTGTCGA
SalI cDNA_intron1
GTCGACCACTGCTGCTTCTTCTGACCAAGAATTCTTTTTCTTTCACTCCACTATTTCATTTTCTTCCC
AAACTTAG
cDNA_exon2-8
ATCTGATCCTGACCAGCACCGCGCCGGAACACCTGAGCGCGCCGACCCTGCCGCTGCCGGAGGTT
CAATGCTTCGTTTTCAACATCGAGTATATGAACTGCACCTGGAACAGCAGCAGCGAGCCGCAGGC
GACCAACCTGACCCTGCACTACCGTTATAAGGTGAGCGACAACAACACCTTCCAAGAGTGCAGCC
ACTACCTGTTTAGCAAGGAAATCACCAGCGGTTGCCAGATCCAAAAAGAAGACATTCAGCTGTAT
CAAACCTTCGTGGTTCAGCTGCAAGATCCGCAGAAGCCGCAACGTCGTGCGGTGCAGAAACTGAA
CCTGCAAAACCTGGTTATTCCGCGTGCGCCGGAGAACCTGACCCTGAGCAACCTGAGCGAGAGCC
AGCTGGAACTGCGTTGGAAGAGCCGTCACATCAAAGAGCGTTGCCTGCAGTACCTGGTGCAATAT
CGTAGCAACCGTGACCGTAGCTGGACCGAGCTGATTGTTAACCACGAACCGCGTTTCAGCCTGCCG
AGCGTGGATGAGCTGAAGCGTTACACCTTTCGTGTTCGTAGCCGTTATAACCCGATCTGCGGTAGC
AGCCAGCAATGGAGCAAATGGAGCCAGCCGGTGCACTGGGGCAGCCACACCGTTGAGGAAAACC
CGAGCCTGTTCGCGCTGGAGGCGGTGCTGATCCCGGTTGGTACCATGGGCCTGATCATTACCCTGA
TTTTCGTGTACTGCTGGCTGGAACGTATGCCGCCGATCCCGCCGATTAAGAACCTGGAGGACCTGG
TGACCGAATATCAGGGCAACTTCAGCGCGTGGAGCGGTGTTAGCAAAGGCCTGACCGAGAGCCTG
CAACCGGATTACAGCGAGCGTTTTTGCCACGTTAGCGAAATTCCGCCGAAAGGTGGCGCGCTGGG
TGAAGGTCCGGGTGGTAGCCCGTGCAGCCTGCATAGCCCGTACTGGCCGCCGCCGTGCTACAGCCT
GAAGCCGGAAGCGTAA
cDNA_polyA
AACTTGTTTATTGCAGCTTATAATGGTTACAAATAAAGCAATAGCATCACAAATTTCACAAATAAA
GCATTTTTTTCACTGCATTCTAGTTGTGGTTTGTCCAAACTCATCAATGTATCTTATCATGTCTGGAT
CCTCGAGCGGCCGCATCGGATCCCTGGCGTAATCATG
M13 reverse
GTCATAGCTGTTTCCTG

Supplemental Tables S1-S5 can be downloaded from a separate Excel file

Supplementary Table S6. gRNA list

**Name**	**gRNA sequence**
m*Il2rg* Intron 1 SpCas9 gRNA 1	GTAGGAAACCTAGGACCAGA-GGG
m*Itga2b* exon 1 SpCas9 gRNA 1	GCACTAGGACCCAAGAACAG-TGG
m*Itga2b* exon 1 SpCas9 gRNA 2	ACTTGCGTTTAGGGCCCTGG-GGG

Supplementary Table 7. Primer list

	Primer	Direction	Sequence	Purpose
1	m*Itgα2b* primer 1F	Forward	GGTGAGCTTTCCTGGAGAGGAAG	Detection of mutation by Surveyor assay at *Itgα2b* site
2	m*Itgα2b* primer 1R	Reverse	TCCACTCAGACCGGAGAACTGAC	Detection of mutation by Surveyor assay at *Itgα2b* site
3	m*Itgα2b* primer 2F	Forward	GACAGGCAGACATTTTGTCTGGT	Detection of mutation by Surveyor assay at *Itgα2b* site
4	m*Itgα2b* primer 2R	Reverse	CTAGAGCAGACCCACAGGAGAG	Detection of mutation by Surveyor assay at *Itgα2b* site
5	m*Il2rg* Intron 1_1F	Forward	GAAAAGGTGGCTGGGAATGATGGT	Detection of mutation by Surveyor assay at *Il2rg* site
6	m*Il2rg* Intron 1_1R	Reverse	AGCTAGCCTCATCTGGTCTGAACT	Detection of mutation by Surveyor assay at *Il2rg* site
7	m*Il2rg*_exon2-4_DS_primer_1F_barcode	Forward	CGCTGATCAAAGGAAATGTATGGGTGGGGAGG	For amplicon sequence to multiplex samples
8	m*Il2rg*_exon2-4_DS_primer_2F_barcode	Forward	AACGTGATAAAGGAAATGTATGGGTGGGGAGG	For amplicon sequence to multiplex samples
9	m*Il2rg*_exon2-4_DS_primer_3F_barcode	Forward	AAACATCGAAAGGAAATGTATGGGTGGGGAGG	For amplicon sequence to multiplex samples
10	m*Il2rg*_exon2-4_DS_primer_4F_barcode	Forward	ATGCCTAAAAAGGAAATGTATGGGTGGGGAGG	For amplicon sequence to multiplex samples
11	m*Il2rg*_exon2-4_DS_primer_1R_barcode	Reverse	ACCACTGTTGTTCAGGGGCTGTAGAAGTCAG	For amplicon sequence to multiplex samples
12	m*Il2rg*_exon2-4_DS_primer_2R_barcode	Reverse	AGTGGTCATGTTCAGGGGCTGTAGAAGTCAG	For amplicon sequence to multiplex samples
13	NHEJ insertion check 1F	Forward	CTGGTGACCGAATATCAGGGCA	To confirm the integration of partial-cDNA at 3'-end
14	NHEJ insertion check 1R	Reverse	CCCTCTGCACACTTCGTTTTAGTCA	To confirm the integration of partial-cDNA at 3'-end
15	NHEJ insertion check 2F	Forward	TCCACCGGAAGCTACGACAAAAG	To confirm the integration of partial-cDNA at 5'-end
16	NHEJ insertion check 2R	Reverse	TGAACCACGAAGGTTTGATACAGCTG	To confirm the integration of partial-cDNA at 5'-end
17	HITI sequence primer 1F	Forward	GCAGATTGTACTGAGAGTGCACCA	To check pUC57-CD132-HITI plasmid sequence
18	HITI sequence primer 1R	Reverse	TCATTAATGCAGCTGGCACGACA	To check pUC57-CD132-HITI plasmid sequence
19	HITI primer 1F	Forward	CTAGGTTTCCTACCCTAGATAGATCTGTCG	To construct HITI sequence at 5'-end of pUC57-CD132 plasmid
20	HITI primer 1R	Reverse	GACCAGAGGGAGTCTAGATGCATTCGCGAG	To construct HITI sequence at 5'-end of pUC57-CD132 plasmid
21	HITI primer 2F	Forward	TAGGTTTCCTACCCGTCGACTGCAGAGGCC	To construct HITI sequence at 3'-end of pUC57-CD132 plasmid
22	HITI primer 2R	Reverse	GGACCAGAGGGAGTCGGGATCCGATGCGGC	To construct HITI sequence at 3'-end of pUC57-CD132 plasmid
