# Supplementary material for: Non-viral ex vivo genome-editing in mouse bona fide hematopoietic stem cells with CRISPR/Cas9
Source: Mol Ther Methods Clin Dev. 2021 Jan 9;20:451–62. doi: 10.1016/j.omtm.2021.01.001 (PMC7873578; doi:10.1016/j.omtm.2021.01.001)
Supplement: Document S3. Article plus supplemental information [file mmc3.pdf]

Molecular Therapy
Methods & Clinical Development
Original Article

Non-viral *ex vivo* genome-editing in mouse bona fide hematopoietic stem cells with CRISPR/Cas9

Suvd Byambaa,[1] Hideki Uosaki,[1] Tsukasa Ohmori,[2] Hiromasa Hara,[1] Hitoshi Endo,[3] Osamu Nureki,[4] and Yutaka Hanazono[1]

[1]Division of Regenerative Medicine, Center for Molecular Medicine, Jichi Medical University, Tochigi 329-0498, Japan; [2]Division of Medical Biochemistry, Department of Biochemistry, School of Medicine, Jichi Medical University, Tochigi 329-0498, Japan; [3]Division of Functional Biochemistry, Department of Biochemistry, School of Medicine, Jichi Medical University, Tochigi 329-0498, Japan; [4]Department of Biological Sciences, Graduate School of Science, The University of Tokyo, Tokyo 113-0033, Japan

We conducted two lines of genome-editing experiments of mouse hematopoietic stem cells (HSCs) with the clustered regularly interspaced short palindromic repeat (CRISPR) and CRISPR-associated protein 9 (Cas9). First, to evaluate the genome-editing efficiency in mouse bona fide HSCs, we knocked out integrin alpha 2b (*Itga2b*) with Cas9 ribonucleoprotein (Cas9/RNP) and performed serial transplantation in mice. The knockout efficiency was estimated at approximately 15%. Second, giving an example of X-linked severe combined immunodeficiency (X-SCID) as a target genetic disease, we showed a proof-of-concept of universal gene correction, allowing rescue of most of X-SCID mutations, in a completely non-viral setting. We inserted partial cDNA of interleukin-2 receptor gamma chain (*Il2rg*) into intron 1 of *Il2rg* via non-homologous end-joining (NHEJ) with Cas9/RNP and a homology-independent targeted integration (HITI)-based construct. Repaired HSCs reconstituted T lymphocytes and thymuses in SCID mice. Our results show that a non-viral genome-editing of HSCs with CRISPR/Cas9 will help cure genetic diseases.

INTRODUCTION

Allogeneic hematopoietic stem cell (HSC) transplantation is the first-line treatment in inherited hematopoietic disorders; however, the availability of human leukocyte antigen (HLA)-matched donors is limited.[1] Gene therapy, a delivery of a gene using gammaretrovirus-derived integrative vectors to autologous HSCs, has shown efficacy in treating X-linked severe combined immunodeficiency (X-SCID), adenosine deaminase deficiency, X-linked adrenoleukodystrophy, β-thalassemia, and Wiskott-Aldrich syndrome.[2–8] X-SCID patients were indeed cured by the therapy; however, the integration sites of therapeutic genes could not be controlled (i.e., random integration occurred), and some insertions, such as one near the LIM domain-only 2 (LMO2) proto-oncogene, caused leukemia.[7–10] Although recent lentiviral gene therapy of X-SCID no longer caused leukemia,[11] the integration site of a transgene is still uncontrollable. Clustered regularly interspaced short palindromic repeat (CRISPR)/Cas9 has opened new possibilities of site-specific insertion of therapeutic genes in human cells, including HSCs.[12,13] Previous studies have shown the feasibility of genome editing in HSCs to treat hematological disorders.[14–16] However, to realize the therapy, there are many issues to be addressed.

Important issues are whether "bona fide" HSCs can be genome edited. Long-term multilineage engraftment of genome-edited HSCs has been shown in nonhuman primate models by some groups.[17–19] Although these models would be the best to assess primate HSCs and presumably the best for translating data into human HSCs, the access to these models is limited. Many researchers are using murine models, and serial transplantation is a widely used standard to assess mouse HSCs; however, it has not been performed to assess the multilineage engraftment of genome-modified mouse HSCs in the past.

For therapeutic genome editing to replace disease-causing mutations with normal ones, homology-directed repair (HDR) with exogenous template DNA, either single-stranded oligodeoxynucleotide (ssODN) or double-stranded DNA, has been widely studied. Inserting cDNA via non-homologous end-joining (NHEJ) is another method under consideration.[20–22] While an NHEJ occurs during the G1 phase, an HDR only occurs during the G2/S phase.[13] As bona fide HSCs mainly stay in the G0/G1 phase,[23,24] HDR-based repair would less likely happen in HSCs.

How to deliver genome-editing tools is another issue to be elucidated. Plasmid transfection,[25] lentiviral vectors,[26] and non-viral Cas9 ribonucleoprotein (Cas9/RNP) methods[13,27–29] are used to edit the HSC genome. Among them, RNP-based methods are getting more

Received 18 November 2020; accepted 6 January 2021;
https://doi.org/10.1016/j.omtm.2021.01.001.

Correspondence: Yutaka Hanazono, MD, PhD, Division of Regenerative Medicine, Center for Molecular Medicine, Jichi Medical University, 3311-1 Yakushiji, Shimotsuke-shi, Tochigi 329-0498, Japan.
E-mail: hanazono@jichi.ac.jp

© 2021 The Authors.
This is an open access article under the CC BY license (http://creativecommons.org/licenses/by/4.0/).

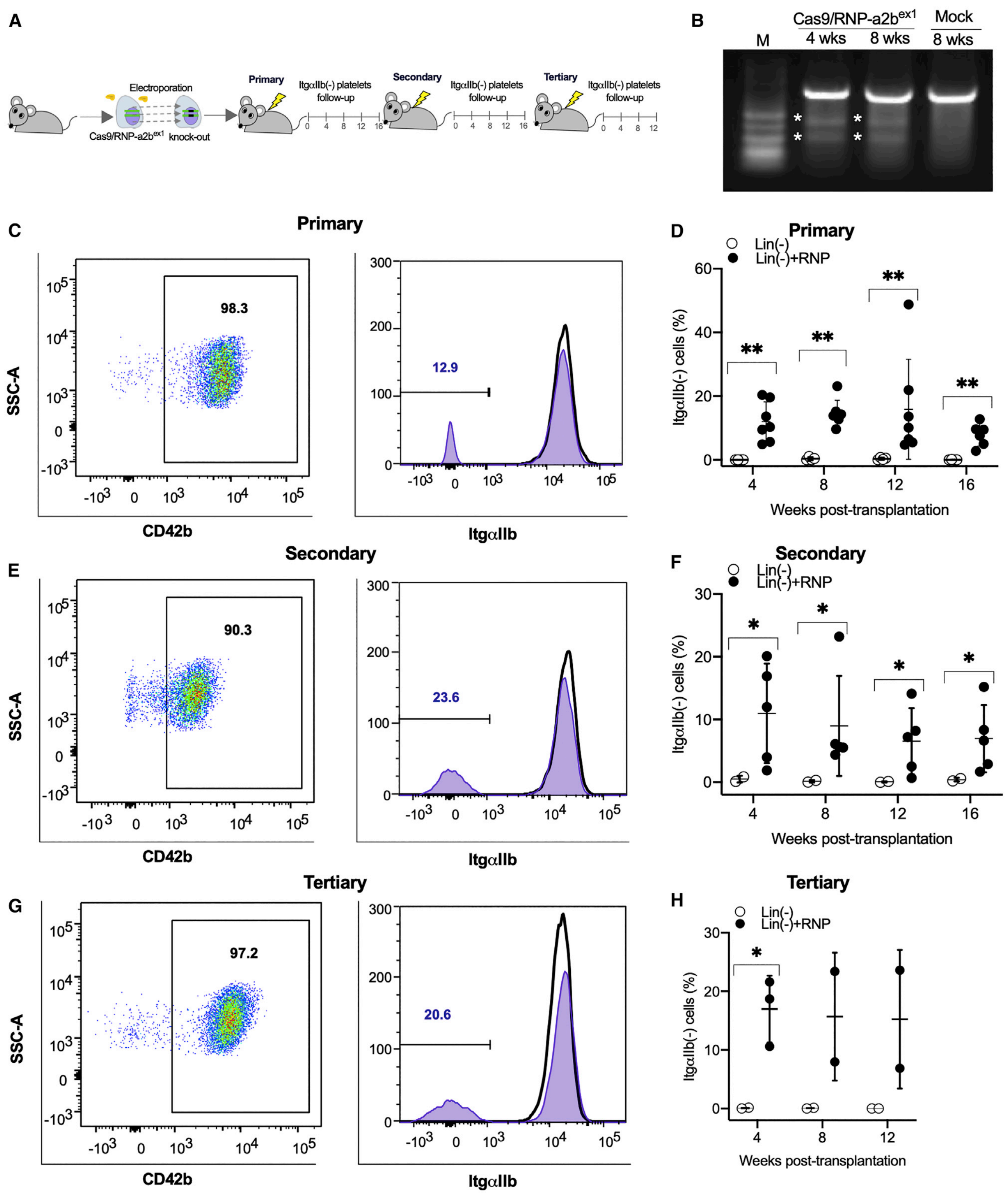

Figure 1. Efficient *Itga2b* knockout in bona fide HSCs

(A) A schematic diagram of serial transplantation of mouse bone marrow Lin⁻ cells (i.e., an HSC-containing fraction), which were electroporated with Cas9/RNP-a2b^{ex1} to lethally irradiated C57BL/6J mice. (B) Surveyor assays of *Itga2b* exon 1 in Lin⁻ cells after transplantation. Asterisks indicate digestion products. (C, E, and G) Representative

(legend continued on next page)

popular, as they are more effective on on-target sites, have lower risk of off-target damages with shorter-lasting exposure of a nuclease, and have lower stimulation of innate immunity than other delivery methods.[30,31]

In this study, we aimed to evaluate genome-editing efficiency in bona fide HSCs by serial transplantation. To avoid viral integration to the HSC genome, we took a non-viral approach to edit the genome in murine HSCs. We first examined knockout efficiency in bona fide HSCs by delivering Cas9/RNP to HSCs *ex vivo* and subsequent serial transplantation. Then, we demonstrated successful NHEJ-based integration of cDNA into mouse HSCs to cure a mouse X-SCID model.

RESULTS

Ex vivo knockout of *Itga2b* gene in bona fide HSCs

It is known that stronger electroporation conditions to deliver Cas9/RNP are more efficient to edit genomes, but damage more cells, and that weaker conditions are gentler to cells but inefficient to edit. Thus, it is important to find an acceptable condition for both genome-editing efficiency and cell viability. To this end, we first optimized the electroporation condition for mouse bone marrow lineage-negative (Lin^-) cells as a fraction of HSCs with the NEPA21 electroporator using green fluorescent protein (GFP) as a reporter. We assessed the delivery efficiency of GFP protein and the viability of cells by GFP fluorescence and 7- amino-actinomycin D (7-AAD) staining, respectively, immediately after electroporation (Figures S1A–S1D). For optimization, we tested poring pulse parameters (voltage, pulse duration, and pulse number; Figures S1B–S1D). Voltages of 75 and 100 V displayed similar efficiencies and cell viabilities. Voltages of more than 100 V shows low viability (Figure S1B). Pulse durations at 3 and 5 ms and a larger number of pulses (8 pulses) exhibited higher efficiency and high viability (Figures S1C and S1D). In this condition (100 V, 5 ms, and 8 pulses), the delivery efficiency of GFP protein was more than 60%, with 50% viability. We further examined pulse parameters in terms of genome-editing efficiency. Consistent with the efficient delivery of GFP protein, mutations at the on-target site were detected with the pulses of 75 and 100 V with Surveyor assay (Figure S1E).

To accurately estimate genome-editing efficiency in bona fide HSCs, we selected *Itga2b* as a target gene and performed serial transplantation after genome editing. *Itga2b* is expressed in megakaryocytes and platelets among blood cells, and the equal ability of *in vivo* hematopoietic reconstitution by either $Itga2b^+$ or $Itga2b^-$ HSCs is reported.[32–34] Moreover, integrin αIIb (ItgαIIb), encoded by *Itga2b*, is a surface marker that can be easily assessed and forms the integrin αIIbβ3 complex mediating platelet adhesion and aggregation.[35] Upon activation, αIIbβ3 becomes detectable on the activated mouse platelets by JON/A antibody, and thus we can see a functional outcome of the genome editing.

First, we introduced the RNP complex of Cas9 and guide RNA (gRNA) targeting *Itga2b* exons 1 and 2 (Cas9/RNP-a2b^{ex1}; Cas9/RNP-a2b^{ex2}) (Figure S2A) to Lin^- cells by electroporation. We detected mutations at both exons 1 and 2 *Itga2b* by Surveyor assay after 7 to 14 days of semi-solid methylcellulose culture *in vitro* (Figure S2B). Next, Lin^- cells after the electroporation of Cas9/RNP-a2b^{ex1} were transplanted to lethally irradiated mice (Figure 1A). The mutation at exon 1 of *Itga2b* was confirmed by Surveyor assay in the peripheral blood of recipient mice after 4 to 8 weeks post-transplantation (Figure 1B). We then examined the proportion of ItgαIIb$^-$ platelets in CD42b$^+$ platelets up to 16 weeks after transplantation (Figures 1C–1H). ItgαIIb$^-$ platelets were 15.8% ± 15.7% (n = 7) at 12 weeks and 7.9% ± 3.6% (n = 6) at 16 weeks after primary transplantation (Figures 1C and 1D). To test if genome editing occurs in bona fide HSCs but not only in progenitors of platelets, we performed serial transplantation of Lin^- cells obtained from the primary recipients. We detected ItgαIIb$^-$ platelets at 6.5% ± 5.2% and at 15.2% ± 11.8% of CD42b$^+$ platelets in secondary (n = 5) (Figures 1E and 1F) and tertiary recipients (n = 3) (Figures 1G and 1H), respectively, at 12 weeks after transplantation. If genome editing occurred in bona fide HSCs, we should be able to detect a site-specific mutation in multi-lineage of blood cells. To test this, we sorted granulocytes and T and B lymphocytes from the bone marrow and peripheral blood of a tertiary recipient after 12 weeks post-transplantation (Figures S2C–S2E). All three lineages of blood cells had mutations in exon 1 of *Itga2b* (Figure S2E). We also performed the same experiment using Lin^- cells derived from GFP-transgenic mice as donor cells to evaluate editing efficiency (Figures S3A and S3B). In this experiment, we observed that ~90% of platelets were derived from the donor through 4 to 12 weeks after transplantation (Figure S3C). There were 5% to 10% of ItgαIIb$^-$ platelets in GFP-positive platelets (Figures S3B and S3D). Taken together, the electroporation of Cas9/RNP can cause a site-specific mutation in the genome of mouse bona fide HSCs at the efficiency of ~15%.

Functional outcome of *Itga2b* disruption

Platelets are activated through contacts with collagen and other molecules, and activated platelets exhibit P-selectin (CD62P) on the plasma membrane from α-granule and aggregates.[36–38] Platelet aggregation is mediated by the active form of integrin αIIbβ3 complex, which can be detected by a specific antibody, JON/A, on mouse platelets.[39] Here, we examined two distinct activation markers of platelets after genome editing (Figure 2A). We stimulated platelets with collagen-related peptide (CRP), which induces conformational change in integrin αIIbβ3 (JON/A binding). We

flow cytometric results showing gating of platelets (positive for CD42B) and successful knockout of ItgαIIb in those platelets isolated from primary (C), secondary (E), and tertiary recipients (G) after serial transplantation of Cas9/RNP-a2b^{ex1}-treated Lin^- cells (open histogram, transplantation of mock Lin^- cells as negative controls; filled-purple histogram, transplantation of Lin^- cells electroporated with Cas9/RNP-a2b^{ex1}). (D, F, and H) Flow cytometric results of ItgαIIb$^-$ platelets in all mice at 4 to 12 weeks after primary to tertiary transplantation showing the knockout efficiency was around 15% even after tertiary transplantation (black circle, transplantation of mock Lin^- cells as negative controls; open circle, transplantation of Lin^- cells electroporated with Cas9/RNP-a2b^{ex1}). Mann-Whitney one-tailed U test: *p < 0.05; **p < 0.01.

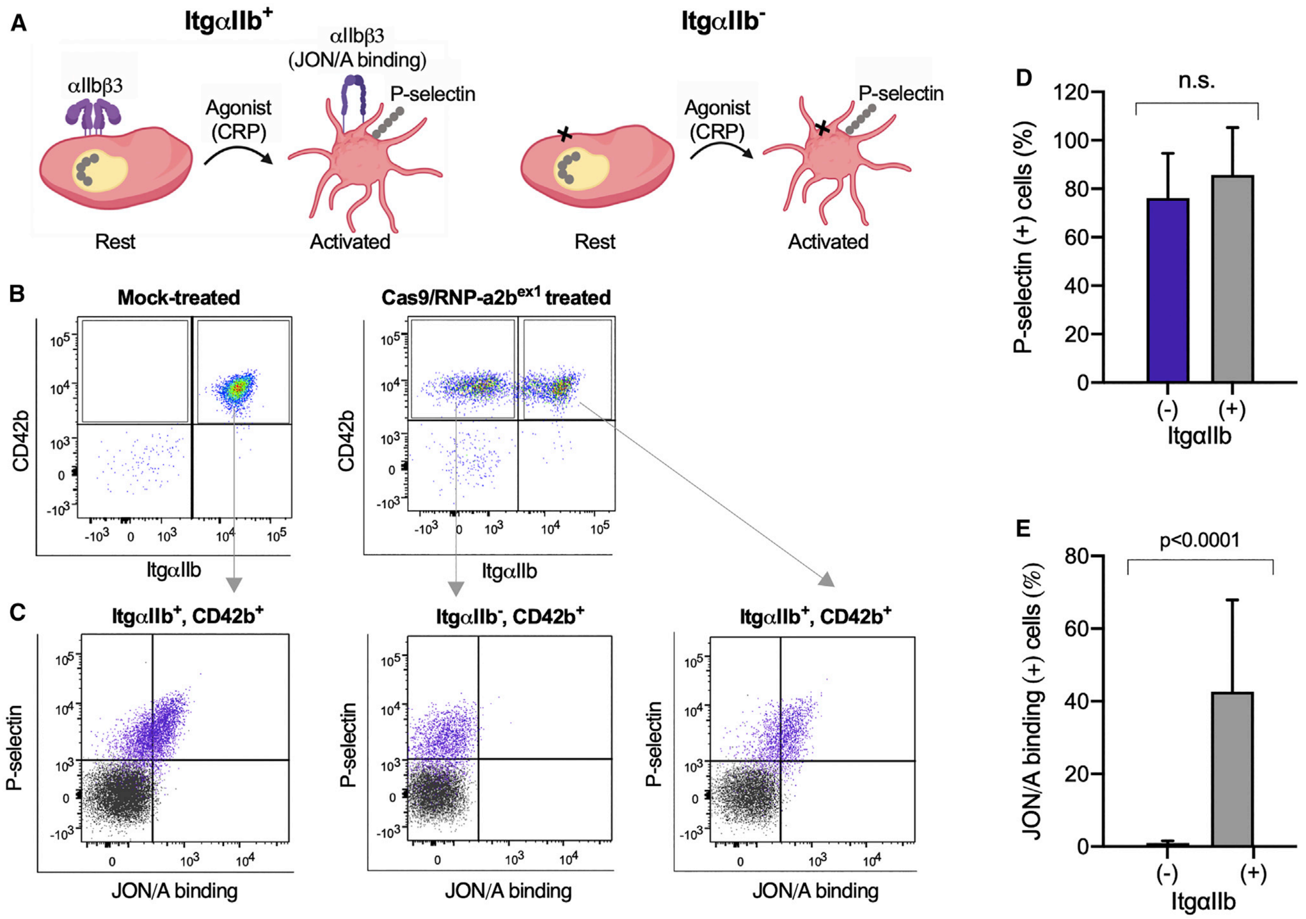

Figure 2. Functional characterization of platelets derived from *Itga2b*-edited HSCs

(A) A schematic representation of platelet activation. Stimulation of platelets with collagen-related peptide (CRP) induces conformational change (activation) in integrin αIIbb3 leading to platelet aggregation, and surface expression of P-selectin (ItgαIIb^{+}). JON/A monoclonal antibody can bind to activated form of integrin αIIbb3. The defect of integrin αIIbb3 by genome editing (ItgαIIb^{-}) on platelets results in inability of aggregation (not to bind to JON/A after activation). (B) Expression of CD42b and integrin αIIb on platelets derived from mock (left) or *Itga2b*-edited HSCs (right). The plots represent the degree of integrin αIIb expression (horizontal) and binding of CD42b (vertical). (C) Activation of integrin αIIbβ3 assessed by JON/A binding (horizontal) and P-selectin expression (vertical) on platelets. Platelets derived from mock (left) or *Itga2b*-edited HSCs (middle and right) were stimulated without (black plot) or with 1 μg/mL of CRP (purple plot). Platelets derived from *Itga2b*-edited HSCs were divided into two groups (i.e., ItgαIIb^{-} [middle] and ItgαIIb^{+} [right]), with respect to αIIb expression, and separately analyzed. The stimulation with CRP elicited the expression of P-selectin, but not activation of integrin αIIbb3, in ItgαIIb^{-} platelets. (D and E) Columns and error bars represent the mean ± SD of P-selectin expression (D) and JON/A binding (E) after stimulation with 1 μg/mL of CRP (n = 6). Blue bars and black bars represent ItgαIIb^{-} and ItgαIIb^{+} platelets derived from *Itga2b*-edited HSCs, respectively. Statistical significance was determined using unpaired t test.

examined P-selectin and JON/A binding on unmodified (ItgαIIb^{+}) or knockout (ItgαIIb^{-}) platelets. Platelets from control mice with the transplantation of mock-treated Lin^{-} cells became positive for P-selectin and JON/A binding after the activation (Figures 2B and 2C). The platelets from the mice after the transplantation of Cas9/RNP-a2b^{ex1}-treated Lin^{-} cells were gated to ItgαIIb^{+} and ItgαIIb^{-} (Figure 2B). After the stimulation with CRP, both ItgαIIb^{+} and ItgαIIb^{-} platelets expressed P-selectin, but the αIIbβ3 was not activated in ItgαIIb^{-} platelets (Figures 2C–2E). These results confirm a functional defect of ItgαIIb^{-} platelets derived from HSCs that were genome edited with Cas9/RNP.

Targeted cDNA integration into intron 1 of *Il2rg* in mouse Lin^{-} cells

For therapeutic genome editing, there is more demand for restoring normal gene function than disrupting a gene. Thus, we next aimed to cure a genetic hematopoietic disease via restoring normal gene function. We chose X-SCID as a target disease. X-SCID is a monogenic disease but has diverse mutations across *IL2RG*. To restore *Il2rg* function in X-SCID mice, we took a strategy of NHEJ- and homology-independent targeted integration (HITI)-based integration of *Il2rg* exon 2–8 cDNA (HITI-cDNAex2-8) into intron 1 of *Il2rg* (Figure 3A) because (1) NHEJ occurs more frequently than HDR when repairing double-strand

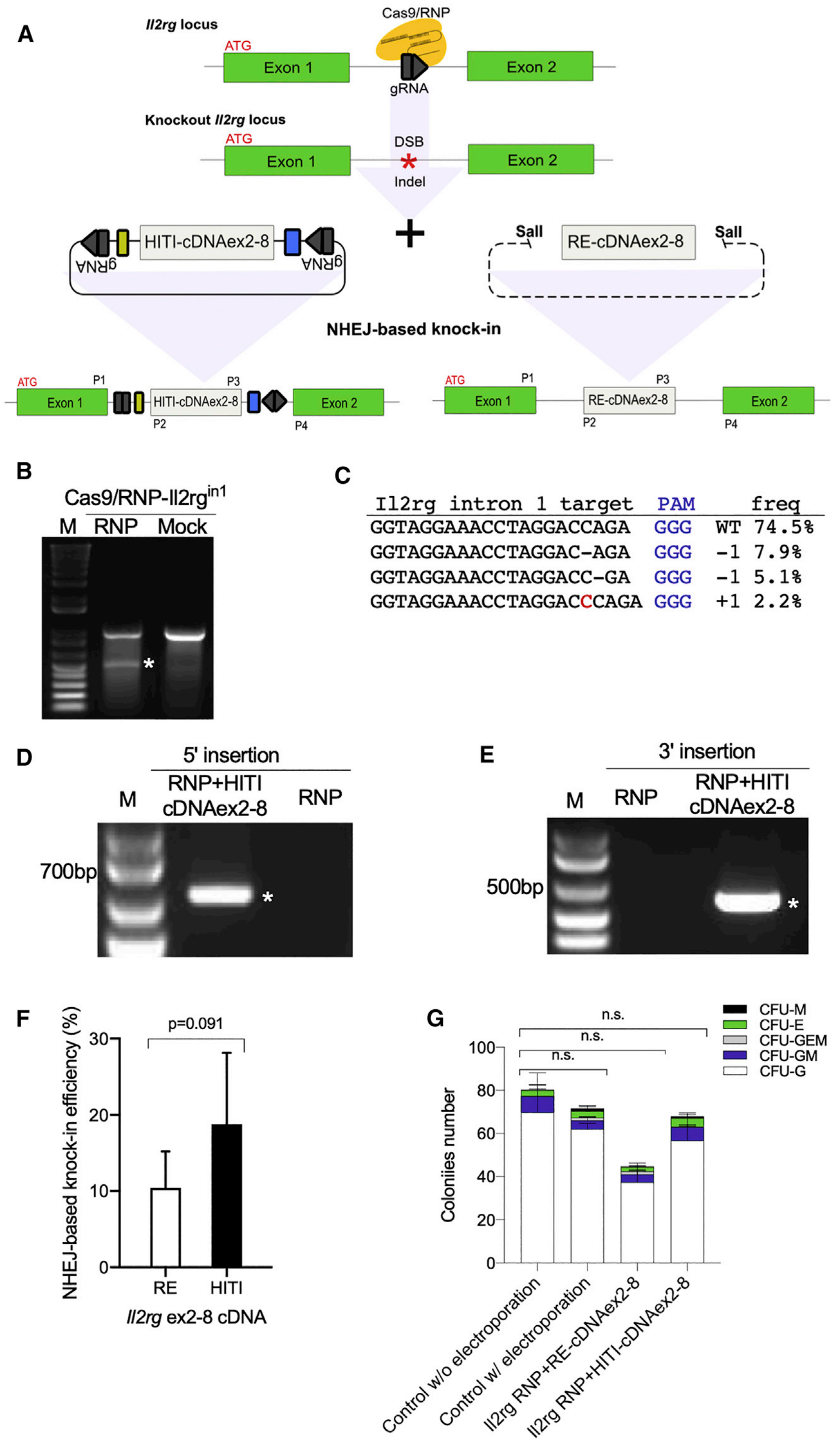

Figure 3. Integration of HITI-cDNAex2-8 into *Il2rg* intron 1 in Lin⁻ cells *in vitro*

(A) A schematic diagram showing the targeted site of gRNAs at the *Il2rg* intron 1 and the integration of HITI-cDNAex2-8 or RE-cDNAex2-8 into *Il2rg* intron 1 via the NHEJ-based knockin (green, *Il2rg* exons; gray, codon-optimized cDNA for *Il2rg* exon2-8; black, HITI sequence [reverse complimented gRNA]; yellow, splice acceptor site; and blue, PolyA). Primer sites are also shown. (B) Surveyor assays at *Il2rg* intron 1 in Lin$^-$ cells at 72 h after electroporation with Cas9/RNP-Il2rg^{in1}. (C) Targeted deep sequencing showing a rate of 20.2% insertions and deletions (indels) in cellular genomes after electroporation with Cas9/RNP-il2rg^{in1} (PAM, purple; mutation, red). (D and E) Genomic PCR results indicating successful knockin of HITI-cDNAex2-8 into intron 1 in Lin$^-$ cells. The 5′-end insertion was confirmed with the P1 + P2 primer set and the 3′-end insertion was confirmed with the P3 + P4 primer set. (F) Knockin efficiency of RE-cDNAex2-8 or HITI-cDNAex2-8 into *Il2rg* intron 1 site in Lin$^-$ cells after Cas9-mediated DSBs were examined by individual clonogenic colony PCR after CFU assay. RE, RE-cDNAex2-8 linearized by restriction enzyme; HITI, HITI-cDNAex2-8 containing HITI-sequence. (G) Absolute numbers of colonies after electroporation of RE-cDNAex2-8 or HITI-cDNAex2-8 to Lin$^-$ cells. Dunnett's multiple comparison test: *$p < 0.05$; **$p < 0.01$.

breaks (DSBs);[22,23] (2) bona fide HSCs are mostly quiescent in the cell cycle, in which NHEJ likely occurs more frequently than HDR; and (3) recently developed HITI has been reported to enhance NHEJ-mediated gene integration with an orientation-directed manner.[40,41] This approach would be universal, as complete *Il2rg* mRNA could be transcribed from the first exon and the inserted partial cDNA of exon 2–8 (Figure 3A), allowing the cure of any X-SCID patients regardless of mutations found in or after the second exon. In addition, this method is meritorious, considering that the natural promoter will function to express *Il2rg*.

First, we tested the method in NIH 3T3 cells (Figure S4). We confirmed that gRNA for *Il2rg* intron 1 with Cas9 (Cas9/RNP-Il2rg^{in1}) introduced by electroporation effectively generated mutations in NIH 3T3 cells (Figures S4A and S4B; Table S1). The site-specific integration of cDNAex2-8 by electroporation of Cas9/RNP-Il2rg^{in1} with HITI-cDNAex2-8 in NIH 3T3 was confirmed by the correct size of PCR products with the primer sets for 5′ and 3′ ends of the inserted sequences (Figure S4C).

Next, we tested mouse bone marrow Lin$^-$ cells in a non-viral setting. We confirmed that the electroporation of Cas9/RNP-Il2rg^{in1} to Lin$^-$ cells caused mutations at 20.2% with Surveyor assay and amplicon sequencing (Figures 3B and 3C; Table S2). To introduce cDNAex2-8 and Cas9/RNP-Il2rg^{in1}, we compared 75 and 100 V, 3 and 5 ms, and 5 and 8 pulse

electroporation conditions (Figures S5A and S5B). Although we observed significant reduction of GFP protein introduction with cDNAex2-8 compared to GFP alone in Lin^- cells (Figures S5A and S5B), the site-specific integration of cDNAex2-8 was confirmed by genomic PCR and sequencing (Figures 3D and 3E; Table S3). We also assessed possible off-target insertions and deletions (indels) induced by Cas9/RNP-$Il2rg^{in1}$. The off-target sites were determined by CCtop or Cas-OFFinder software (Tables S4 and S5). By CCTop, we could not find off-targets with fewer than 3 mismatches. With Cas-OFFinder, we found 8 putative off-target sites with 2 mismatches and 1 bulge. Among them, three were located in exon (Table S5). Putative off-target sites were then analyzed in RNP+HITI-cDNAex2-8-treated Lin^- cells by Surveyor assay; however, no specific mutations were observed (Figure S5C).

HITI allows efficient insertion with an orientation-directed manner via NHEJ in neurons and other cell types through re-cutting of the wrong directional insertion.[40] To confirm HITI functions in Lin^- cells, we compared two forms of cDNAex2-8, cDNAex2-8 linearized by restriction enzyme (RE-cDNAex2-8) or cDNAex2-8 plus HITI sequence (HITI-cDNAex2-8) in the presence of Cas9/RNP-$Il2rg^{in1}$. To calculate knockin efficiency accurately, we cultured electroporated Lin^- cells for colony-forming unit (CFU) assay, then examined the integration in each of the single colonies by PCR. We found that HITI-cDNAex2-8 tends to improve nearly double the knockin efficiency as compared to the RE-cDNAex2-8 as expected (that is, 18.7% ± 14.3% versus 10.3% ± 11.0%) (Figure 3F). We also counted colony numbers after the electroporation, and there was no significant difference in colony numbers between the electroporation of HITI-cDNAex2-8 and mock Lin^- cells (Figure 3G), indicating that the genome-editing procedure did not alter the colony-forming ability of genome-edited Lin^- cells. Taken together, we successfully performed the targeted knockin of *Il2rg* exon 2-8 cDNA into *Il2rg* intron 1 of HSCs in a non-viral setting.

Phenotypical correction of *Il2rg*-mutated mice after transplantation of genome-edited HSCs

We examined if this targeted knockin could cure X-SCID phenotypes *in vivo*. Specifically, we electroporated the Cas9/RNP-$Il2rg^{in1}$ with or without the therapeutic construct (HITI-cDNAex2-8) to bone marrow Lin^- cells of *Il2rg*-mutated mice (C57BL/6 background, CD45.2) that were generated previously and then transplanted the treated cells to another SCID strain, NODShi.Cg-$Prkdc^{scid}Il2rg^{tm1Sug}$ (NOG) mice (CD45.1), to distinguish donor and host cells in the allogeneic transplantation (Figure 4A). Three out of 13 primary recipients showed increases in white blood cell counts and development of T and B cells by 8 weeks after transplantation, considered as cured (Figures 4B–4D; Figures S6A and S6B). In the cured mice, donor-derived $CD45.2^+$ $CD3^+$ T cells (13.2% ± 10.5%) and $CD45.2^+$ $CD19^+$ B cells (9.3% ± 11.7%) at 12 weeks after transplantation and $CD45.2^+$ $CD3^+$ T cells (5.6% ± 4.9%) and $CD45.2^+$ $CD19^+$ B cells (29.7% ± 38.3%) were detected as early as 6 weeks after transplantation (Figures 4C and 4D; Figure S6). In contrast, T cells were hardly observed in non-cured and control mice (<0.5%) (Figure 4C; Figure S6A). Furthermore, donor-derived $CD45.2^+$ $CD3^+$ T cells expressed *Il2rg* in the peripheral blood, bone marrow, and thymus of the cured mice (Figure 4E). The integration of HITI-cDNAex2-8 in intron 1 was confirmed by genomic PCR (Figure S7A). The symptoms of graft-versus-host disease (Figure S7B) were also observed, suggesting that the reconstituted T lymphocytes were functional *in vivo*. Notably, we observed the development of thymus in one cured recipient at 20 weeks after transplantation (Figure 4F), while thymus was not observed in a control recipient (Figure S7C). We then performed secondary transplantation from the cured mice to *Il2rg*-mutated mice. In the secondary recipients, T and B cells were reconstituted, and T cells expressed Il2rg in peripheral blood, bone marrow, and thymus at 12 weeks post-transplantation (Figure 4G; Figures S7D–S7F). These results indicate that partial cDNA insertion to an intron can restore the gene expression under the natural promotor regardless of where mutations exist in the *Il2rg* exons and rescue X-SCID mice when the repaired HSCs are successfully engrafted.

DISCUSSION

In this study, we demonstrated that CRISPR/Cas9-mediated knockout and partial cDNA integration occurred in bona fide HSCs in a completely non-viral setting. Cas9/RNP delivery via electroporation efficiently caused mutations (around 15%) in bona fide HSCs that were previously believed to be difficult targets.[42] We also successfully demonstrated a universal gene correction of X-SCID mutations in mouse HSCs leading to the cure of the mice.

To ensure bona fide HSCs were edited, we showed the detection of DSB at *Itgα2b* not only in myeloid and megakaryocytic lineages but also in T and B cells derived from tertiary recipient mice (Figure S2E). We estimated the gene-editing (*Itgα2b*-knockout) efficiency of bona fide HSCs from the serial transplantation experiments. The gene-knockout efficiency of the peripheral blood cells would be reflected by the gene-knockout efficiency in bona fide (long-term) HSCs. It would also be reflected by short-term HSCs and repopulating progenitor cells; however, when you repeat replating the cells into subsequent mice (i.e., serial transplantation), short-term HSCs and repopulating progenitor cells would no longer repopulate adequately in tertiary recipient mice.[43] Therefore, the gene knockout efficiency of the peripheral blood cells of the tertiary recipient mice would faithfully be reflected only by bona fide (long-term) HSCs. As shown in Figure 1H, the gene knockout efficiency of the peripheral blood platelets in the tertiary recipient mice was around 15%. Those were the grounds on which we estimated the gene-editing efficiency in bona fide HSCs at 15%.

The universal gene correction, consisting of Cas9/RNP and a HITI-containing partial cDNA construct, effectively integrated the cDNA into the target intron by NHEJ-based repair following Cas9-mediated DSBs. In addition, as compared with the HDR-based repair method, NHEJ-based partial cDNA insertion has another advantage. This method can be used for any type of mutation if the mutations locate after the target intron, whereas HDR needs to be optimized and assessed for each mutation. Furthermore, we integrated the HITI

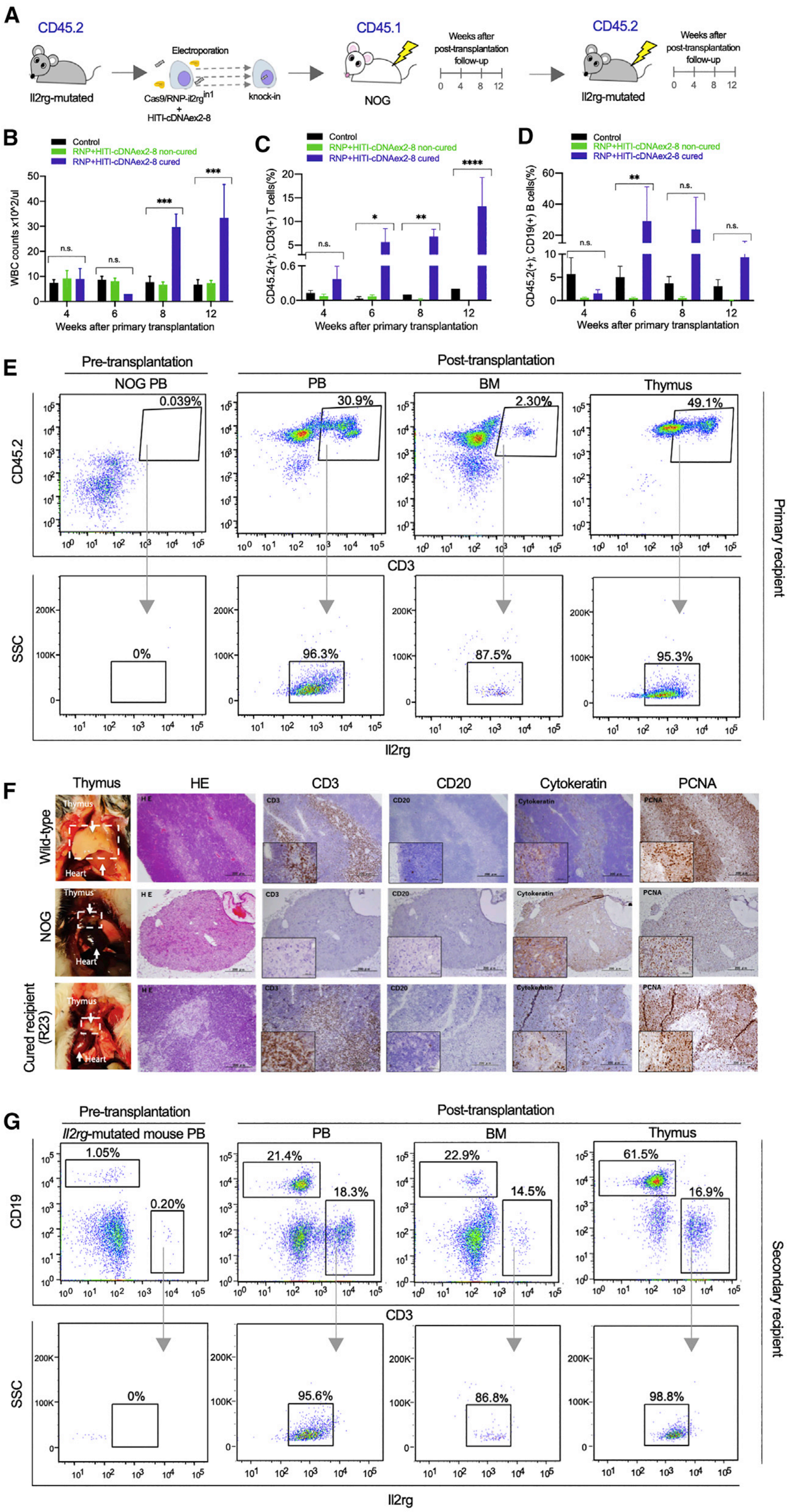

Figure 4. Reconstitution of T/B cells and thymus after non-viral genome editing

(A) A schematic diagram of proof-of-concept experiments of genome-editing therapy for X-SCID. Lin^- cells of *Il2rg*-mutated mice (CD45.2) were electroporated with the therapeutic construct and Cas9/RNP-$Il2rg^{in1}$ or with Cas9/RNP-$Il2rg^{in1}$ alone as a control and transplanted to NOG mice (CD45.1). Lin^- cells (CD45.1/2) of cured NOG were transplanted serially to *Il2rg*-mutated mice (CD45.2). (B–D) White blood cell counts (B), and the fractions of $CD3^+$ T cells (C) and $CD19^+$ B cells (D) among donor-derived $CD45.2^+$ cells in the control, non-cured, and cured recipients (black, controls; green, non-cured among those receiving the therapeutic construct; violet, cured among those receiving the therapeutic construct). ANOVA Tukey's test (*$p < 0.05$; **$p < 0.01$; ***$p < 0.001$). (E and G) Representative flow cytometric results of the peripheral blood, bone marrow, and thymus after the primary (F) and secondary transplantation (G) of genome-edited Lin^- cells. (F) Detection of donor-derived $CD45.2^+$ and $CD3^+$ T cells (upper panels) and Il2rg among the donor-derived T cells (lower panels) in primary recipient mice. (G) Detection of $CD3^+$ T and $CD19^+$ B cells (upper panels) and Il2rg among $CD3^+$ T cells (lower panels) in secondary recipient mice. (F) Macroscopic and microscopic observation of thymuses from wild-type, NOG, and a cured recipient (R23). The thymus of the cured recipient was smaller than the wild type; however, it evidently developed from a NOG mouse (1st column). Comparative histology of thymuses stained with hematoxylin and eosin (2nd column) and stained with antibodies against CD3, CD20, cytokeratin, and PCNA (3rd to 6th columns). Scale bars, 200 μm (50 μm in insets). $CD3^+$, $cytokeratin^+$, and $PCNA^+$ with a few $CD20^+$ cells were detected in the thymus of the cured recipient (R23), though it still lacked Hassall's corpuscles that are usually present in the wild-type thymus. In contrast, NOG mice lacked all of $CD3^+$ cells, $CD20^+$ cells, and Hassall's corpuscles, and most cells were $cytokeratin^+$ in the traces of thymuses.

method to insert cDNA in an orientation-directed manner to improve the efficiency of the NHEJ-based method.[40]

Our results indicate that electroporation of Cas9/RNP to HSCs can be performed without using any viral vectors. Genome-editing technologies have provided a possibility for site-specific modification of genes, but they still need improvement in terms of accuracy and efficacy.[44] Non-integrating viruses such as adenoviruses or adeno-associated viruses (AAVs) are often used to deliver CRISPR/Cas9, but they could evoke immune responses against constitutively expressed Cas9 in hosts.[45,46] In contrast, Cas9/RNP is rapidly degraded in transduced cells, which evades immune responses in hosts and, more importantly, generates fewer off-target DSBs than other delivery methods.[47–49] We also estimated possible off-target sites using off-target detection software such as Cas-OFFinder and CCTop[50,51] and found no putative off-target sites with fewer than three mismatches using CCTop; there are eight sites with 2-bp mismatch and 1-bp bulge by Cas-OFFinder. In addition, we could not observe any specific Surveyor band for these putative off-target sites. Therefore, we anticipate that the gRNA we used in this study would not cause any off-target damages to coding sequences, as DSBs unlikely occur on sequences with two or more mismatches with Cas9/RNP.

Some issues remain to be addressed in future studies. First, there is still a room to improve the genome-editing efficiency in bona fide HSCs. It was ~15% for the knockout in this study. Some reports suggested that genome-editing efficiency assessed *in vivo* after transplantation became lower than that assessed *in vitro* before transplantation.[52] One of the reasons for this discrepancy is that bona fide HSCs are sensitive to Cas9-mediated genome editing.[52] Whether they are tolerant or sensitive to Cas9, HSCs responsible for hematopoietic reconstitution are generally considered a tougher target for genome editing than hematopoietic progenitor cells such as Lin^- cells.[53] Although the integration method of partial cDNA plus HITI worked, the overall efficiency should be improved, given that only 0.08% of Lin^- cells are bona fide HSCs[54] and HITI-based integration occurs only 10% of DSBs.[55,56] We also observed a significantly reduced protein introduction with DNA compared to protein alone (Figures S5A and S5B), which might limit the successful integration to only a few cells per million Lin^- cells. Second, when we analyzed the integration site at 5′ and 3′ ends by Sanger sequencing, 20–40 bp deletions from the putative Cas9 cleavage sites were found (Table S3), although HITI-based integration was reported to integrate cDNA without such a deletion. A 25-bp deletion shaved 7 bp from exon 1. In this case, frameshift and/or splice donor site deletion occurred, and expression should be altered, which might also limit successful cases. Moreover, we need to evaluate if the expression can be fully restored after partial cDNA insertion into intron 1 of *Il2rg*. In our previous study of partial cDNA insertion by NHEJ or HDR in hepatocytes, we observed less expression from NHEJ-based insertion than from HDR-based insertion.[1] Thus, increasing the efficiency of the genome editing and gene integration and the delivery method of Cas9/RNP with an integration vector is required for the future successful treatment of hematopoietic diseases.

To avoiding viral integration to the HSC genome, another possible delivery method would be the use of non-integrating AAV vectors. AAV-based genome editing has shown successful insertion of therapeutic genes or repair of genomic mutations with or without Cas9.[57–59] However, when an AAV vector is used to express Cas9 in non-dividing cells, an immune response may be evoked against Cas9, given that the AAV vector is retained in a cell for a long period when cells are not dividing. In addition, the AAV vector may cause unwanted integration as lentivirus did, as the AAV vector will happen to integrate into the genome in the presence of CRISPR-Cas9.[60] Therefore, it is important to evaluate the efficiency and safety carefully. In the present study, we have not compared the HITI-based construct with the HDR-based or non-HITI NHEJ methods, such as 2-hit-2-oligo.[61,62] It remains to be determined if the HITI method is truly superior to other methods.

In conclusion, we demonstrated that the electroporation with NHEJ-based insertion of partial cDNA into an intron combined with Cas9-RNP and HITI-based donor plasmid is a promising strategy to recapture the correct gene expression under the control of its natural promoter without the use of viral vectors, unlike authentic gene therapies. Although further studies are required, this would pave the way to cure genetic diseases such as X-SCID.

MATERIAL AND METHODS

Animals

Il2rg-mutated mice with a one-nucleotide insertion in *Il2rg* exon 4 were generated previously using C57BL/6J mice[63] and C57BL/6J mice were purchased from SLC Japan (Shizuoka, Japan). NOG mice (6 weeks old), a SCID strain, were obtained from the Central Institute for Experimental Animals (Kawasaki, Japan). GFP-transgenic mice (B6 Tg:CAG-GFP105) were generated at Jichi Medical University. All animal experiments reported here were approved by the Institutional Animal Care and Concern Committee at Jichi Medical University. Animal care and all experiments were performed under the committee's guidelines.

Preparation of mouse Lin^- cells

Total bone marrow cells were isolated from the tibias and femurs of 6- to 10-week-old male C57B6/J and *Il2rg*-mutated mice. Then, the cells were treated with ammonium-chloride-potassium (ACK) lysing buffer to remove erythrocytes, followed by magnetic bead selection of Lin^- fractions using antibodies against Gr-1, CD11b, B220 (CD45R), Ter119, and CD3 (BioLegend, San Diego, CA, USA). Isolated Lin^- cells were stimulated for 24 h in Iscove's modified Dulbecco's medium (IMDM) medium supplemented with insulin–transferrin–selenium (ITS) supplement-A (STEMCELL Technologies, Vancouver, BC, Canada), 100 ng/mL murine thrombopoietin (BioLegend), 100 ng/mL stem cell factor (BioLegend), and 100 ng/mL Flt3-ligand (BioLegend) before electroporation.

Preparation of Cas9/RNP and delivery of Cas9/RNP and cDNA

We designed gRNA for the *Il2rg* intron 1 site and *Itga2b* exons 1 and 2 (gRNA list in Table S6) using online software by Benchling (https://benchling.com/faq).

Recombinant *S. pyogenes* Cas9 protein containing three N-terminal nuclear localization sequences (NLS) (Alt-R S.p. Cas9 Nuclease 3NLS) was purchased from Integrated DNA Technologies (Skokie, IL, USA). Universal trans-activating CRISPR RNA (crRNA) (tracrRNA) (Alt-R CRISPR-Cas9 tracrRNA) and target-specific crRNA (Alt-R CRISPR-Cas9 crRNA) were also purchased from Integrated DNA Technologies. Purified recombinant GFP (rAcGFP1) was purchased from Takara Bio (Kyoto, Japan).

For Lin^- cells, we optimized electroporation conditions to 100 V, 5 ms, and 8 pulses for the poring pulse and 10 V, 50 ms, and 5 pulses for the transfer pulse using NEPA21 Super Electroporator (Nepagene, Chiba, Japan). To prepare a Cas9/RNP complex, an equimolar concentration of a target-specific crRNA (100 μM) and tracrRNA (100 μM) were incubated for 5 min at 95°C and cooled down to room temperature to form a crRNA:tracrRNA duplex. Then, the target-specific crRNA:tracrRNA duplex (60 μM) and Cas9 protein (61 μM) were mixed in OPTI-MEM (Thermo Fisher Scientific, Waltham, MA, USA) and further incubated for 20 min at room temperature. Freshly prepared Cas9/RNP was electroporated into 2×10^5 Lin^- cells as described above. To integrate *Il2rg* exon 2–8 cDNA, we prepared two plasmids containing *Il2rg* exon 2–8 cDNA with HITI (HITI-cDNAex2-8) or SalI recognition sites at the 5′ and 3′ ends of the cDNA. RE-cDNAex2-8 was prepared from a plasmid vector by digestion with SalI and purified by gel extraction of the specific size using the QIAquick Gel Extraction kit (QIAGEN, Hilden, Germany). One microgram of RE-cDNAex2-8 fragment or HITI-cDNAex2-8 plasmid (Figure 3A) was electroporated simultaneously with Cas9/RNP-$Il2rg^{in1}$ at the conditions of 100 V, 5 ms, and 8 pulses for the poring pulse and 10 V, 50 ms, and 5 pulses for the transfer pulse using NEPA21 Super Electroporator and cuvettes with a 1 mm gap.

Serial transplantation

For the transplantation of *Itga2b*-knockout cells, Lin^- cells were collected and electroporated with Cas9/RNP-$a2b^{ex1}$ on the same day. The cells after electroporation were recovered and cultured with IMDM medium (supplemented with ITS supplement-A, 100 ng/mL murine thrombopoietin, 100 ng/mL stem cell factor, and 100 ng/mL Flt3-ligand) for 2 to 6 h. Then, the cells were washed 3 times with PBS before transplantation. The cells ($0.3–1 \times 10^6$) were resuspended in PBS with 0.5% BSA and 2 mM EDTA and injected via a jugular vein into 6- to 8-week-old lethally irradiated (9.5 Gy) C57BL/6J mice with CellRad X (Precision X-Ray, North Branford, CT, USA) or Gamma cell-40 machine (MDS Nordion, Ottawa, ON, Canada). For secondary and tertiary transplantation, whole bone marrow cells were collected from the recipients at 12 to 16 weeks after the primary and secondary transplantation, respectively. Then, the cells (1×10^7) were injected via a jugular vein into lethally irradiated C57BL/6J mice.

For the transplantation of *Il2rg* knockin cells, Lin^- cells were collected from *Il2rg*-mutated mice and electroporated with Cas9/RNP and HITI-cDNAex2-8 on the same day of electroporation. The cells after electroporation were handled following the same procedure as mentioned above. The cells ($0.4–1.2 \times 10^6$) were resuspended in PBS with 0.5% BSA and 2 mM EDTA and injected via a jugular vein into 6- to 8-week-old sublethally irradiated (2.5 Gy) NOG mice with CellRad X. The irradiated mice were treated with drinking water supplemented with neomycin (1 mg/mL) for 4 weeks to prevent infections while the mice were immunocompromised. For the secondary and tertiary transplantation, whole bone marrow cells were collected from the recipients at 12 to 16 weeks after the primary and secondary transplantation, respectively. Then, the cells (1×10^7) were injected via a jugular vein into lethally irradiated (9.5 Gy) *Il2rg*-mutated mice with CellRad X or Gamma cell-40 machine.

Whole blood (30 μL) per mouse was collected via a jugular vein every 4 weeks after transplantation, and complete blood cell counts were performed with the Celltac-alpha machine (Nihon Kohden, Tokyo, Japan).

Surveyor nuclease assay and knockin confirmation

CRISPR/Cas9-mediated genomic mutations were detected with the Surveyor mutation detection kit (Integrated DNA Technologies). The target sites of *Itga2b* exons 1 and 2 and *Il2rg* (primers in Table S7) were amplified with ExTaq DNA polymerase (Takara Bio, Otsu, Japan), and heteroduplex was formed under the denaturing and re-annealing protocol with a thermal cycler as indicated in the manufacturer's instructions. Then, samples were treated with Surveyor nuclease and DNA fragments were analyzed by agarose gel electrophoresis. For the putative off-target sites, each genomic region was amplified with the primer shown in Table S5, and Surveyor assay was performed as described above.

To analyze in detail the mutations detected in the Surveyor assays, DNA sequencing was performed. A PCR fragment of each genomic region was amplified, cloned to the pTAC-2 vector (TA PCR Cloning Kit, Biodynamics), and then transformed to One Shot Stbl3 Chemically Competent *E. coli* (Thermo Fisher Scientific). Plasmids were extracted by the QIAprep Spin Miniprep kit (QIAGEN, Hilden, Germany), and DNA sequencing was performed for each clone.

To confirm the integration of partial cDNA into intron 1 of the *Il2rg* gene, we performed genomic PCR for 5′- and 3′-end integration sites. The 5′-end and 3′-end insertion was confirmed by PCR with primers in Table S7. Then, samples were analyzed by agarose gel electrophoresis or MultiNA, a microchip electrophoresis system (Shimadzu, Tokyo, Japan) with DNA-1000 Reagent Kit (Shimadzu, Tokyo, Japan).

Methylcellulose CFU assay

Two days after Cas9/RNP electroporation, cells were plated on 35-mm dishes with mouse MethylCult medium (STEMCELL Technologies, Vancouver, BC, Canada). After 10–14 days, colonies were counted according to their morphological features for CFU of granulocytes (CFU-G), granulocytes and monocytes (CFU-GM), erythroid (CFU-E), monocytes (CFU-M), and mixed (CFU-GEMM). To examine knockin efficiency of cDNAex2-8 into *Il2rg* intron 1 with

or without HITI, 96 well-separated, individual colonies were picked up, and PCR of each colony was performed by 5′-end integration (expected 622bp PCR product) and 3′ end integration (expected 466bp PCR product), respectively (primers in Table S7).

Immunohistological staining

Thymuses were immunostained with mouse anti-human CD3 monoclonal antibody (clone F7.2.38, Agilent, Santa Clara, CA, USA), mouse anti-human CD20cy monoclonal antibody (clone L26, Agilent), mouse anti-human cytokeratin monoclonal antibody (clone AE1/AE3, Agilent), and mouse anti-proliferating cell nuclear antigen monoclonal antibody (clone PC10, Agilent) as primary antibodies and with Enbision+ Dual Link System-HRP:peroxidase-labeled polymer (Agilent) as a secondary antibody.

Blood collection and preparation of washed platelets

Mice were anesthetized with isoflurane, and a blood sample (50–200 μL) was collected by heparinized syringes through a jugular vein. Whole blood was diluted by 3 mL of HEPES/Tyrode's buffer (138 mmol/L NaCl, 3.3 mmol/L NaH_2PO_4, 2.9 mmol/L KCl, 1 mmol/L $MgCl_2$, 1 mg/mL glucose, and 20 mmol/L HEPES, pH7.4). The diluted blood was centrifuged at 100 × *g* for 10 min. The platelet-rich fraction was resuspended in HEPES/Tyrode's buffer and centrifuged at 2,200 × g for 10 min. The pellet containing platelets was washed with HEPES/Tyrode's buffer. To prevent the activation of platelets, HEPES/Tyrode's buffer was added with 1 μg/mL prostaglandin (Funakoshi, Tokyo, Japan) before the procedure. For analysis of platelet activation, washed platelets were activated with 1 U/mL of thrombin or 1 μg/mL CRP.

Flow cytometry

Peripheral blood was obtained through a jugular vein and mononuclear cells were isolated through erythrocyte lysis. The cells were stained with anti-mouse CD45-fluorescein isothiocyanate (FITC) (BD PharMingen), anti-CD3-PE, anti-CD19-PE/Cy7, anti-CD11b-BV421, anti-Gr-1-BV510, and anti-CD132-APC (BioLegend) to identify lymphoid and myeloid lineages. Washed and activated platelets were stained with Brilliant Violet 421-labeled anti-mouse CD41 (BioLegend), DyLight 649-labeled rat anti-mouse GPIbα (CD42b), phycoerythrin (PE)-labeled anti-activated integrin αIIbβ3 (JON/A), and FITC-labeled anti-mouse CD62P (Anti-P-Selectin) (Emfret Analytics, Eibelstadt, Germany) antibodies. Flow cytometry was performed with BD LSRFortessa II.

Amplicon sequencing

The target sites of *Il2rg* intron 1 site were amplified with a KAPA HiFi HotStart PCR kit (KAPA Biosystems, Willington, MA, USA). To multiplex samples, we used barcoded primers in the Table S7. PCR products were purified with AMPure XP beads (Beckman Coulter, Brea, CA, USA). PCR amplicons were sent to Hokkaido System Science for amplicon sequencing. Illumina sequencing adaptors with indices were added to the amplicons, and the library was subjected to 300 paired-end read sequencing with Illumina MiSeq (100,000 reads). To analyze the data, sixty base pairs near the target were extracted, and the frequency of each sequence was calculated (Tables S1 and S2). The amplicon sequencing data have been deposited in the Sequence Read Archive (PRJNA596061).

Statistics

Statistical analysis of data from all experiments was performed using GraphPad Prism version 8. These statistical data are presented as means ± SD. Significance analyses were performed with two-way ANOVA Tukey's test (*p < 0.05; **p < 0.01; ***p < 0.001) or Mann-Whitney one-tailed t test (*p < 0.05; **p < 0.01; ***p < 0.001).

SUPPLEMENTAL INFORMATION

Supplemental Information can be found online at https://doi.org/10.1016/j.omtm.2021.01.001.

ACKNOWLEDGMENTS

We thank Hiroko Hayakawa for expert technical support for flow cytometry. We also appreciate Tomoyuki Abe, Hiroaki Shibata, Tamaki Aoki, Mika Kishimoto, Yaeko Sutoh, Eri Noguchi, Nawin Chanthra, Rie Ishihara, and Mari Karube for their technical assistance and helpful discussion. This study was supported in part by the Japan Agency for Medical Research and Development (AMED; JP18am0301002 to O.N. and JP20ae0201007 to Y.H. as co-PI) and the Ministry of Education, Culture, Sports, Science and Technology (MEXT; JPS1311030 and JPMXP0620319815 to Y.H.). The purchases of BD LSR Fortessa II, BD Fusion, and MultiNA were supported by JKA through its promotion funds from KEIRIN RACE.

AUTHOR CONTRIBUTIONS

Funding acquisition, supervision, investigation and writing – review & editing, O.N., T.O., and Y.H.; conceptualization, data curation, formal analysis, methodology and writing – original draft, S.B. and H.U.; resources and writing – review & editing, H.E. and H.H.

DECLARATION OF INTERESTS

The authors declare no competing interests.

REFERENCES

1. Antoine, C., Müller, S., Cant, A., Cavazzana-Calvo, M., Veys, P., Vossen, J., Fasth, A., Heilmann, C., Wulffraat, N., Seger, R., et al.; European Group for Blood and Marrow Transplantation; European Society for Immunodeficiency (2003). Long-term survival and transplantation of haemopoietic stem cells for immunodeficiencies: report of the European experience 1968-99. Lancet *361*, 553–560.

2. Aiuti, A., Slavin, S., Aker, M., Ficara, F., Deola, S., Mortellaro, A., Morecki, S., Andolfi, G., Tabucchi, A., Carlucci, F., et al. (2002). Correction of ADA-SCID by stem cell gene therapy combined with nonmyeloablative conditioning. Science *296*, 2410–2413.

3. Cartier, N., Hacein-Bey-Abina, S., Bartholomae, C.C., Veres, G., Schmidt, M., Kutschera, I., Vidaud, M., Abel, U., Dal-Cortivo, L., Caccavelli, L., et al. (2009). Hematopoietic stem cell gene therapy with a lentiviral vector in X-linked adrenoleukodystrophy. Science *326*, 818–823.

4. Braun, C.J., Boztug, K., Paruzynski, A., Witzel, M., Schwarzer, A., Rothe, M., Modlich, U., Beier, R., Göhring, G., Steinemann, D., et al. (2014). Gene therapy for Wiskott-Aldrich syndrome–long-term efficacy and genotoxicity. Sci. Transl. Med. *6*, 227ra33.

5. Cavazzana-Calvo, M., Hacein-Bey, S., de Saint Basile, G., Gross, F., Yvon, E., Nusbaum, P., Selz, F., Hue, C., Certain, S., Casanova, J.L., et al. (2000). Gene therapy of human severe combined immunodeficiency (SCID)-X1 disease. Science *288*, 669–672.

6. Cavazzana-Calvo, M., Payen, E., Negre, O., Wang, G., Hehir, K., Fusil, F., Down, J., Denaro, M., Brady, T., Westerman, K., et al. (2010). Transfusion independence and HMGA2 activation after gene therapy of human β-thalassaemia. Nature *467*, 318–322.

7. Hacein-Bey-Abina, S., Von Kalle, C., Schmidt, M., McCormack, M.P., Wulffraat, N., Leboulch, P., Lim, A., Osborne, C.S., Pawliuk, R., Morillon, E., et al. (2003). LMO2-associated clonal T cell proliferation in two patients after gene therapy for SCID-X1. Science *302*, 415–419.

8. Hacein-Bey-Abina, S., Garrigue, A., Wang, G.P., Soulier, J., Lim, A., Morillon, E., Clappier, E., Caccavelli, L., Delabesse, E., Beldjord, K., et al. (2008). Insertional oncogenesis in 4 patients after retrovirus-mediated gene therapy of SCID-X1. J. Clin. Invest. *118*, 3132–3142.

9. Baum, C., von Kalle, C., Staal, F.J.T., Li, Z., Fehse, B., Schmidt, M., Weerkamp, F., Karlsson, S., Wagemaker, G., and Williams, D.A. (2004). Chance or necessity? Insertional mutagenesis in gene therapy and its consequences. Mol. Ther. *9*, 5–13.

10. Howe, S.J., Mansour, M.R., Schwarzwaelder, K., Bartholomae, C., Hubank, M., Kempski, H., Brugman, M.H., Pike-Overzet, K., Chatters, S.J., de Ridder, D., et al. (2008). Insertional mutagenesis combined with acquired somatic mutations causes leukemogenesis following gene therapy of SCID-X1 patients. J. Clin. Invest. *118*, 3143–3150.

11. Mamcarz, E., Zhou, S., Lockey, T., Abdelsamed, H., Cross, S.J., Kang, G., Ma, Z., Condori, J., Dowdy, J., Triplett, B., et al. (2019). Lentiviral Gene Therapy Combined with Low-Dose Busulfan in Infants with SCID-X1. N. Engl. J. Med. *380*, 1525–1534.

12. Gundry, M.C., Brunetti, L., Lin, A., Mayle, A.E., Kitano, A., Wagner, D., Hsu, J.I., Hoegenauer, K.A., Rooney, C.M., Goodell, M.A., and Nakada, D. (2016). Highly Efficient Genome Editing of Murine and Human Hematopoietic Progenitor Cells by CRISPR/Cas9. Cell Rep. *17*, 1453–1461.

13. Dever, D.P., Bak, R.O., Reinisch, A., Camarena, J., Washington, G., Nicolas, C.E., Pavel-Dinu, M., Saxena, N., Wilkens, A.B., Mantri, S., et al. (2016). CRISPR/Cas9 β-globin gene targeting in human haematopoietic stem cells. Nature *539*, 384–389.

14. Lattanzi, A., Meneghini, V., Pavani, G., Amor, F., Ramadier, S., Felix, T., Antoniani, C., Masson, C., Alibeu, O., Lee, C., et al. (2019). Optimization of CRISPR/Cas9 Delivery to Human Hematopoietic Stem and Progenitor Cells for Therapeutic Genomic Rearrangements. Mol. Ther. *27*, 137–150.

15. Tothova, Z., Krill-Burger, J.M., Popova, K.D., Landers, C.C., Sievers, Q.L., Yudovich, D., Belizaire, R., Aster, J.C., Morgan, E.A., Tsherniak, A., and Ebert, B.L. (2017). Multiplex CRISPR/Cas9-Based Genome Editing in Human Hematopoietic Stem Cells Models Clonal Hematopoiesis and Myeloid Neoplasia. Cell Stem Cell *21*, 547–555.e8.

16. Wu, Y., Zeng, J., Roscoe, B.P., Liu, P., Yao, Q., Lazzarotto, C.R., Clement, K., Cole, M.A., Luk, K., Baricordi, C., et al. (2019). Highly efficient therapeutic gene editing of human hematopoietic stem cells. Nat. Med. *25*, 776–783.

17. Yu, K.R., Dunbar, C.E., Corat, M., Chen, S., Aljanahi, A., Baek, E.J., Metais, J.Y., Natanson, H., Winkler, T., and Donahue, R.E. (2017). A Non-Human Primate CRISPR/Cas9 Model of Clonal Hematopoiesis of Indeterminate Potential Demonstrates Expansion of TET2-Disrupted Clones. Blood *130*, 117.

18. Humbert, O., Radtke, S., Samuelson, C., Carrillo, R.R., Perez, A.M., Reddy, S.S., Lux, C., Pattabhi, S., Schefter, L.E., Negre, O., et al. (2019). Therapeutically relevant engraftment of a CRISPR-Cas9-edited HSC-enriched population with HbF reactivation in nonhuman primates. Sci. Transl. Med. *11*, eaaw3768.

19. Demirci, S., Zeng, J., Wu, Y., Uchida, N., Shen, A.H., Pellin, D., Gamer, J., Yapundich, M., Drysdale, C., Bonanno, J., et al. (2020). BCL11A enhancer-edited hematopoietic stem cells persist in rhesus monkeys without toxicity. J. Clin. Invest. *130*, 6677–6687.

20. Cox, D.B.T., Platt, R.J., and Zhang, F. (2015). Therapeutic genome editing: prospects and challenges. Nat. Med. *21*, 121–131.

21. Román-Rodríguez, F.J., Ugalde, L., Álvarez, L., Díez, B., Ramírez, M.J., Risueño, C., Cortón, M., Bogliolo, M., Bernal, S., March, F., et al. (2019). NHEJ-Mediated Repair of CRISPR-Cas9-Induced DNA Breaks Efficiently Corrects Mutations in HSPCs from Patients with Fanconi Anemia. Cell Stem Cell *25*, 607–621.e7.

22. He, X., Tan, C., Wang, F., Wang, Y., Zhou, R., Cui, D., You, W., Zhao, H., Ren, J., and Feng, B. (2016). Knock-in of large reporter genes in human cells via CRISPR/Cas9-induced homology-dependent and independent DNA repair. Nucleic Acids Res. *44*, e85.

23. Ohmori, T., Nagao, Y., Mizukami, H., Sakata, A., Muramatsu, S.I., Ozawa, K., Tominaga, S.I., Hanazono, Y., Nishimura, S., Nureki, O., and Sakata, Y. (2017). CRISPR/Cas9-mediated genome editing via postnatal administration of AAV vector cures haemophilia B mice. Sci. Rep. *7*, 4159.

24. Maruyama, T., Dougan, S.K., Truttmann, M.C., Bilate, A.M., Ingram, J.R., and Ploegh, H.L. (2015). Increasing the efficiency of precise genome editing with CRISPR-Cas9 by inhibition of nonhomologous end joining. Nat. Biotechnol. *33*, 538–542.

25. Mohrin, M., Bourke, E., Alexander, D., Warr, M.R., Barry-Holson, K., Le Beau, M.M., Morrison, C.G., and Passegué, E. (2010). Hematopoietic stem cell quiescence promotes error-prone DNA repair and mutagenesis. Cell Stem Cell *7*, 174–185.

26. Heckl, D., Kowalczyk, M.S., Yudovich, D., Belizaire, R., Puram, R.V., McConkey, M.E., Thielke, A., Aster, J.C., Regev, A., and Ebert, B.L. (2014). Generation of mouse models of myeloid malignancy with combinatorial genetic lesions using CRISPR-Cas9 genome editing. Nat. Biotechnol. *32*, 941–946.

27. Mandal, P.K., Ferreira, L.M.R., Collins, R., Meissner, T.B., Boutwell, C.L., Friesen, M., Vrbanac, V., Garrison, B.S., Stortchevoi, A., Bryder, D., et al. (2014). Efficient ablation of genes in human hematopoietic stem and effector cells using CRISPR/Cas9. Cell Stem Cell *15*, 643–652.

28. Holt, N., Wang, J., Kim, K., Friedman, G., Wang, X., Taupin, V., Crooks, G.M., Kohn, D.B., Gregory, P.D., Holmes, M.C., and Cannon, P.M. (2010). Human hematopoietic stem/progenitor cells modified by zinc-finger nucleases targeted to CCR5 control HIV-1 in vivo. Nat. Biotechnol. *28*, 839–847.

29. Dever, D.P., and Porteus, M.H. (2017). The changing landscape of gene editing in hematopoietic stem cells: a step towards Cas9 clinical translation. Curr. Opin. Hematol. *24*, 481–488.

30. Zeng, J., Wu, Y., Ren, C., Bonanno, J., Shen, A.H., Shea, D., Gehrke, J.M., Clement, K., Luk, K., Yao, Q., et al. (2020). Therapeutic base editing of human hematopoietic stem cells. Nat. Med. *26*, 535–541.

31. Drysdale, C.M., Tisdale, J.F., and Uchida, N. (2019). Immunoresponse to Gene-Modified Hematopoietic Stem Cells. Mol. Ther. Methods Clin. Dev. *16*, 42–49.

32. Wang, J., Liu, Z., Zhang, S., Wang, X., Bai, H., Xie, M., Dong, F., and Ema, H. (2019). Lineage marker expression on mouse hematopoietic stem cells. Exp. Hematol. *76*, 13–23.e2.

33. Nakorn, T.N., Miyamoto, T., and Weissman, I.L. (2003). Characterization of mouse clonogenic megakaryocyte progenitors. Proc. Natl. Acad. Sci. USA *100*, 205–210.

34. Kiel, M.J., Yilmaz, Ö.H., Iwashita, T., Yilmaz, O.H., Terhorst, C., and Morrison, S.J. (2005). SLAM family receptors distinguish hematopoietic stem and progenitor cells and reveal endothelial niches for stem cells. Cell *121*, 1109–1121.

35. Gekas, C., and Graf, T. (2013). CD41 expression marks myeloid-biased adult hematopoietic stem cells and increases with age. Blood *121*, 4463–4472.

36. Jurk, K., and Kehrel, B.E. (2005). Platelets: physiology and biochemistry. Semin. Thromb. Hemost. *31*, 381–392.

37. Poon, M.-C., Di Minno, G., d'Oiron, R., and Zotz, R. (2016). New Insights Into the Treatment of Glanzmann Thrombasthenia. Transfus. Med. Rev. *30*, 92–99.

38. Frenette, P.S., Johnson, R.C., Hynes, R.O., and Wagner, D.D. (1995). Platelets roll on stimulated endothelium in vivo: an interaction mediated by endothelial P-selectin. Proc. Natl. Acad. Sci. USA *92*, 7450–7454.

39. Huang, J., Li, X., Shi, X., Zhu, M., Wang, J., Huang, S., Huang, X., Wang, H., Li, L., Deng, H., et al. (2019). Platelet integrin αIIbβ3: signal transduction, regulation, and its therapeutic targeting. J. Hematol. Oncol. *12*, 26.

40. Suzuki, K., Tsunekawa, Y., Hernandez-Benitez, R., Wu, J., Zhu, J., Kim, E.J., Hatanaka, F., Yamamoto, M., Araoka, T., Li, Z., et al. (2016). In vivo genome editing via CRISPR/Cas9 mediated homology-independent targeted integration. Nature *540*, 144–149.

41. Nakade, S., Tsubota, T., Sakane, Y., Kume, S., Sakamoto, N., Obara, M., Daimon, T., Sezutsu, H., Yamamoto, T., Sakuma, T., and Suzuki, K.T. (2014). Microhomology-mediated end-joining-dependent integration of donor DNA in cells and animals using TALENs and CRISPR/Cas9. Nat. Commun. *5*, 5560.

42. Nagree, M.S., López-Vásquez, L., and Medin, J.A. (2015). Towards in vivo amplification: Overcoming hurdles in the use of hematopoietic stem cells in transplantation and gene therapy. World J. Stem Cells *7*, 1233–1250.

43. Yamamoto, R., Morita, Y., Ooehara, J., Hamanaka, S., Onodera, M., Rudolph, K.L., Ema, H., and Nakauchi, H. (2013). Clonal analysis unveils self-renewing lineage-restricted progenitors generated directly from hematopoietic stem cells. Cell *154*, 1112–1126.

44. Li, H., Yang, Y., Hong, W., Huang, M., Wu, M., and Zhao, X. (2020). Applications of genome editing technology in the targeted therapy of human diseases: mechanisms, advances and prospects. Signal Transduct. Target. Ther. *5*, 1.

45. Chew, W.L., Tabebordbar, M., Cheng, J.K.W., Mali, P., Wu, E.Y., Ng, A.H.M., Zhu, K., Wagers, A.J., and Church, G.M. (2016). A multifunctional AAV-CRISPR-Cas9 and its host response. Nat. Methods *13*, 868–874.

46. Wang, D., Mou, H., Li, S., Li, Y., Hough, S., Tran, K., Li, J., Yin, H., Anderson, D.G., Sontheimer, E.J., et al. (2015). Adenovirus-Mediated Somatic Genome Editing of *Pten* by CRISPR/Cas9 in Mouse Liver in Spite of Cas9-Specific Immune Responses. Hum. Gene Ther. *26*, 432–442.

47. DeWitt, M., Magis, W., Bray, N.L., Wang, T., Berman, J.R., Urbinati, F., Muñoz, D.P., Kohn, D.B., Walters, M.C., Carroll, D., et al. (2016). Efficient Correction of the Sickle Mutation in Human Hematopoietic Stem Cells Using a Cas9 Ribonucleoprotein Complex. bioRxiv, doi: 10.1101/.

48. Liang, X., Potter, J., Kumar, S., Zou, Y., Quintanilla, R., Sridharan, M., Carte, J., Chen, W., Roark, N., Ranganathan, S., et al. (2015). Rapid and highly efficient mammalian cell engineering via Cas9 protein transfection. J. Biotechnol. *208*, 44–53.

49. Kim, S., Kim, D., Cho, S.W., Kim, J., and Kim, J.-S. (2014). Highly efficient RNA-guided genome editing in human cells via delivery of purified Cas9 ribonucleoproteins. Genome Res. *24*, 1012–1019.

50. Stemmer, M., Thumberger, T., Del Sol Keyer, M., Wittbrodt, J., and Mateo, J.L. (2015). CCTop: An Intuitive, Flexible and Reliable CRISPR/Cas9 Target Prediction Tool. PLoS ONE *10*, e0124633.

51. Bae, S., Park, J., and Kim, J.-S. (2014). Cas-OFFinder: a fast and versatile algorithm that searches for potential off-target sites of Cas9 RNA-guided endonucleases. Bioinformatics *30*, 1473–1475.

52. Schiroli, G., Ferrari, S., Conway, A., Jacob, A., Capo, V., Albano, L., Plati, T., Castiello, M.C., Sanvito, F., Gennery, A.R., et al. (2017). Preclinical modeling highlights the therapeutic potential of hematopoietic stem cell gene editing for correction of SCID-X1. Sci. Transl. Med. *9*, eaan0820.

53. Genovese, P., Schiroli, G., Escobar, G., Tomaso, T.D., Firrito, C., Calabria, A., Moi, D., Mazzieri, R., Bonini, C., Holmes, M.C., et al. (2014). Targeted genome editing in human repopulating haematopoietic stem cells. Nature *510*, 235–240.

54. Osawa, M., Nakamura, K., Nishi, N., Takahasi, N., Tokuomoto, Y., Inoue, H., and Nakauchi, H. (1996). In vivo self-renewal of c-Kit+ Sca-1+ Lin(low/-) hemopoietic stem cells. J. Immunol. *156*, 3207–3214.

55. Nami, F., Basiri, M., Satarian, L., Curtiss, C., Baharvand, H., and Verfaillie, C. (2018). Strategies for In Vivo Genome Editing in Nondividing Cells. Trends Biotechnol. *36*, 770–786.

56. Li, H., Haurigot, V., Doyon, Y., Li, T., Wong, S.Y., Bhagwat, A.S., Malani, N., Anguela, X.M., Sharma, R., Ivanciu, L., et al. (2011). In vivo genome editing restores haemostasis in a mouse model of haemophilia. Nature *475*, 217–221.

57. Schiroli, G., Conti, A., Ferrari, S., Della Volpe, L., Jacob, A., Albano, L., Beretta, S., Calabria, A., Vavassori, V., Gasparini, P., et al. (2019). Precise Gene Editing Preserves Hematopoietic Stem Cell Function following Transient p53-Mediated DNA Damage Response. Cell Stem Cell *24*, 551–565.e8.

58. Kuo, C.Y., Long, J.D., Campo-Fernandez, B., de Oliveira, S., Cooper, A.R., Romero, Z., Hoban, M.D., Joglekar, A.V., Lill, G.R., Kaufman, M.L., et al. (2018). Site-Specific Gene Editing of Human Hematopoietic Stem Cells for X-Linked Hyper-IgM Syndrome. Cell Rep. *23*, 2606–2616.

59. Hiramoto, T., Li, L.B., Funk, S.E., Hirata, R.K., and Russell, D.W. (2018). Nuclease-free Adeno-Associated Virus-Mediated Il2rg Gene Editing in X-SCID Mice. Mol. Ther. *26*, 1255–1265.

60. Hanlon, K.S., Kleinstiver, B.P., Garcia, S.P., Zaborowski, M.P., Volak, A., Spirig, S.E., Muller, A., Sousa, A.A., Tsai, S.Q., Bengtsson, N.E., et al. (2019). High levels of AAV vector integration into CRISPR-induced DNA breaks. Nat. Commun. *10*, 4439.

61. Yoshimi, K., Kunihiro, Y., Kaneko, T., Nagahora, H., Voigt, B., and Mashimo, T. (2016). ssODN-mediated knock-in with CRISPR-Cas for large genomic regions in zygotes. Nat. Commun. *7*, 10431.

62. Sakata, A., Ohmori, T., Nishimura, S., Suzuki, H., Madoiwa, S., Mimuro, J., Kario, K., and Sakata, Y. (2014). Paxillin is an intrinsic negative regulator of platelet activation in mice. Thromb. J. *12*, 1.

63. Byambaa, S., Uosaki, H., Hara, H., Nagao, Y., Abe, T., Shibata, H., Nureki, O., Ohmori, T., and Hanazono, Y. (2020). Generation of novel *Il2rg*-knockout mice with clustered regularly interspaced short palindromic repeats (CRISPR) and Cas9. Exp. Anim. *69*, 189–198.

OMTM, Volume *20*

Supplemental Information

Non-viral *ex vivo* genome-editing in mouse bona fide hematopoietic stem cells with CRISPR/Cas9

Suvd Byambaa, Hideki Uosaki, Tsukasa Ohmori, Hiromasa Hara, Hitoshi Endo, Osamu Nureki, and Yutaka Hanazono

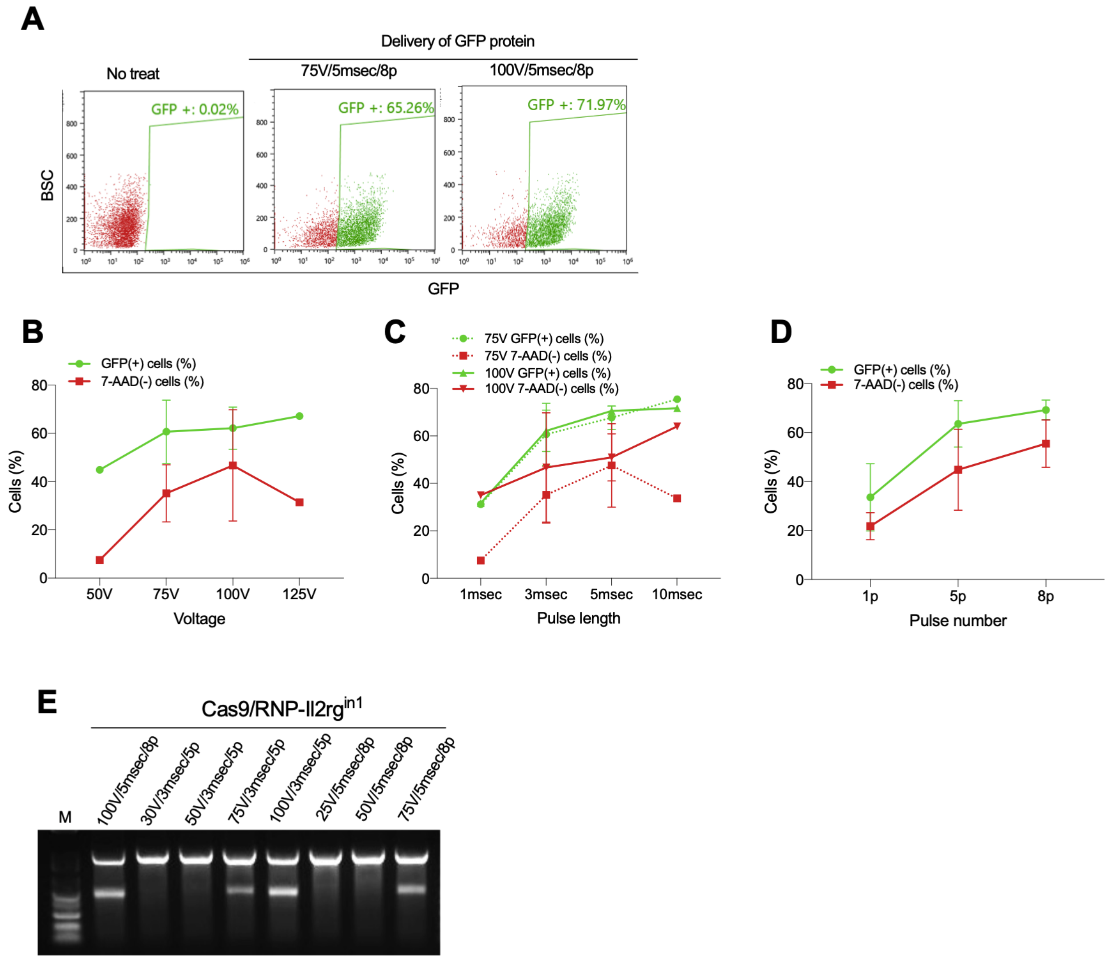

Figure S1. The electroporation conditions were optimized by delivery with GFP protein.

A. Delivery of GFP as a protein to Lin$^-$ cells were assessed by flow cytometry. The conditions of 75 V or 100 V, 5 msec, and 8 pulses for the poring pulse; and those of 10 V, 50 ms, and 5 pulses for the transfer pulse were optimal.

B - D. Optimization of electroporation conditions in Lin$^-$ cells with GFP protein using NEPA21 Super Electroporator. The efficiency of GFP delivery (green) and cell viability (red) were analyzed by flow cytometry. We compared poring pulse parameters: voltage (B), 50 to 125 V with 5 msec and 8 pulses; pulse duration (C), 1 to 10 msec with 100 V and 8 pulses; and pulse number (D), 1 to 8 pulses with 100 V and 5 msec.

E. Surveyor assays at *Il2rg* intron 1 in Lin$^-$ cells at 72 hours after different electroporation conditions to examine DSB at the target site.

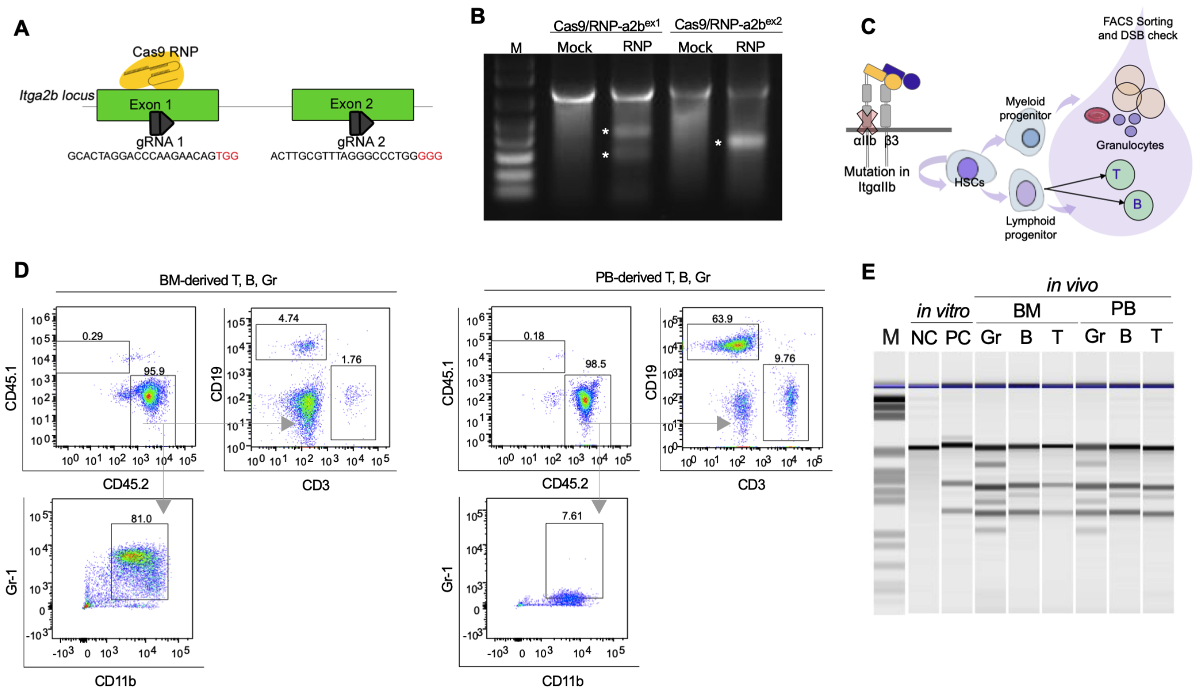

Figure S2. In vitro genome-editing of *Itga2b* in Lin⁻ cells and in vivo detection of the knockout of *Itga2b* from flow cytometry sorted peripheral blood and bone marrow-derived granulocytes and T and B lymphocytes from tertiary recipient.

A. A schematic diagram of the target site in the exon 1 and 2 of the mouse *Itga2b* gene locus.

B. We introduced Cas9/RNP-a2b^{ex1} and Cas9/RNP-a2b^{ex2} to mouse bone marrow Lin^{-} cells by electroporation, and the mutations were examined by Surveyor assay after 5-day culture in vitro.

C. A schematic diagram of knockout of *Itga2b* gene in mouse HSC and flow cytometric sorting of HSC-derived granulocytes and T and B lymphocytes from the bone marrow and peripheral blood of recipients.

D. The detection of mutations in exon 1 of *Itga2b* in sorted granulocytes and T and B lymphocytes from a tertiary recipient after 12 weeks post-transplantation. BM, bone marrow; PB, peripheral blood; Gr, granulocytes; B, B lymphocytes; and T, T lymphocytes; NC, negative control; PC, positive control. PC was the same sample used in Fig 2b that had a site-specific cleavage in vitro.

E. The detection of mutations in exon 1 of *Itga2b* in sorted granulocytes and T and B lymphocytes from a tertiary recipient after 12 weeks post-transplantation.

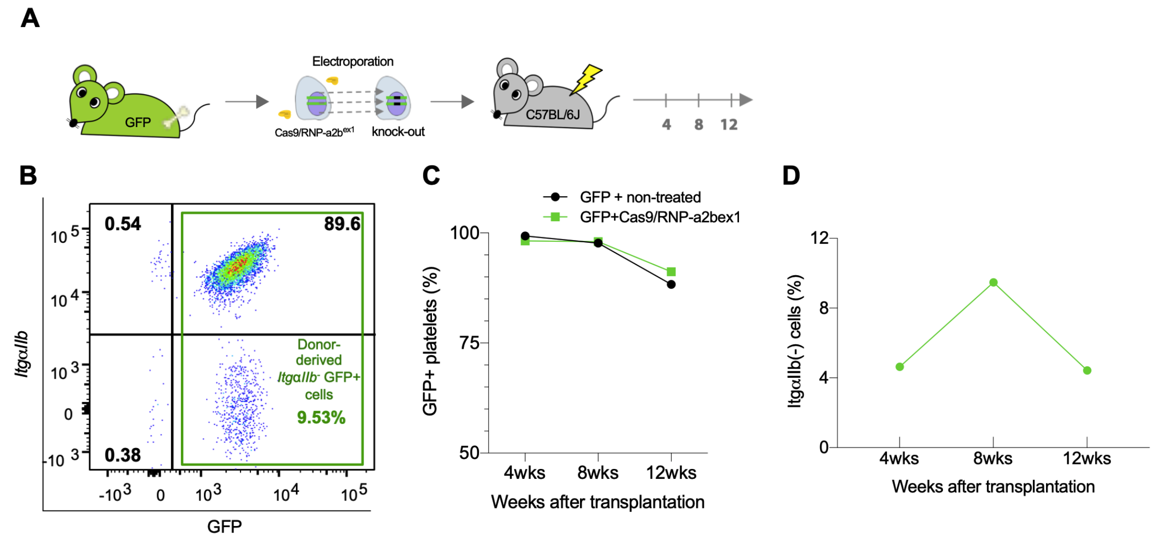

Figure S3. Genome-editing of *Itga2b* using GFP^+ Lin^- cells.

A. A schematic diagram of transplantation of GFP^+ Lin^- cells after electroporation with Cas9/RNP-a2b^{ex1}.

B. Flow cytometric results of donor-derived (GFP^+) ItgαIIb$^-$ platelets at 8 weeks post-transplantation.

C. More than 90% of platelets were positive for GFP; that is, donor-derived.

D. Around 10% of GFP^+ platelets were ItgαIIb$^-$; that is, successfully genome-edited donor HSC-derived.

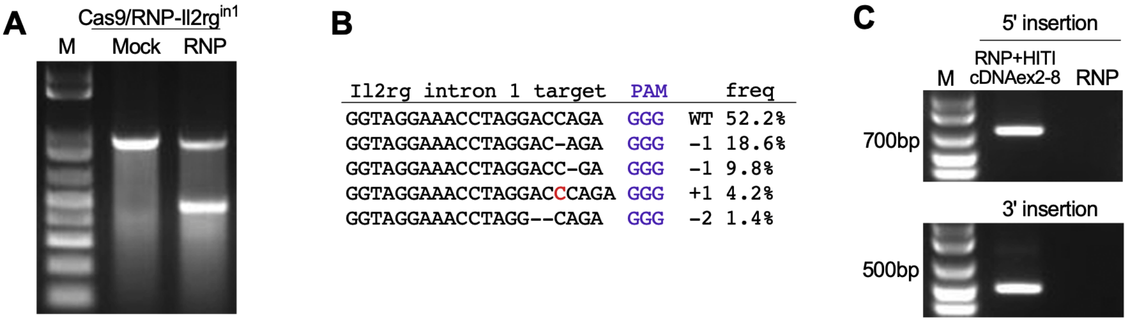

Figure S4. Integration of HITI-cDNAex2-8 into *Il2rg* intron 1 in NIH3T3 cells in vitro.

A. Surveyor assays at *Il2rg* intron 1 in NIH3T3 at 72 hours after electroporation with Cas9/RNP-Il2rg^{in1}.

B. Targeted deep sequencing showing a rate of 43.1% insertions and deletions (InDels) in the cellular genomes after electroporation with Cas9/RNP-il2rg^{in1} (PAM, purple; mutations, red).

C. Genomic PCR results indicating successful knock-in of HITI-cDNA ex2-8 into intron 1 in NIH3T3 cells. The 5'-end insertion was confirmed with the P1+P2 primer set and the 3'-end insertion was confirmed with the P3+P4 primer set.

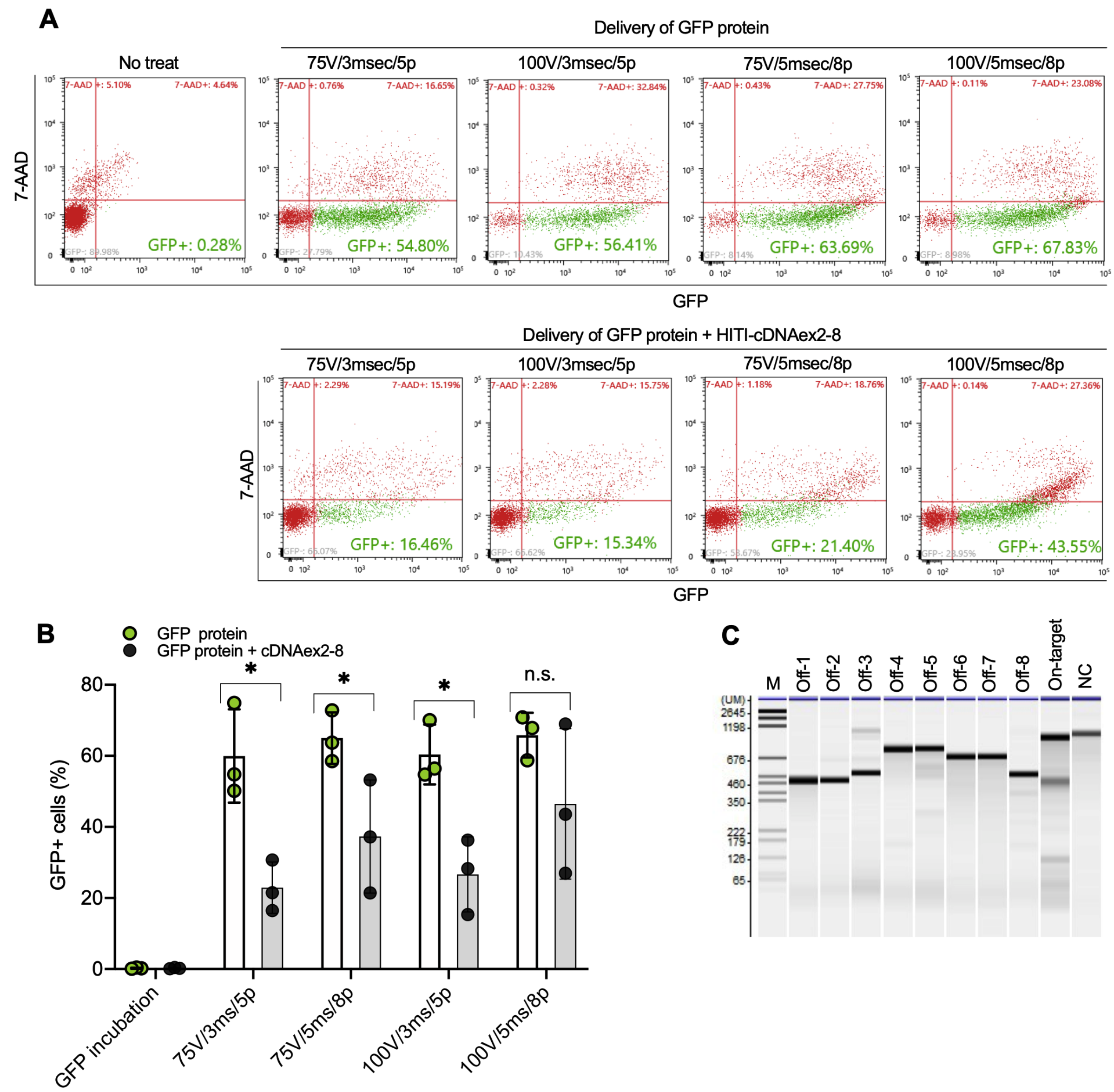

Figure S5. A. Representative plots of flow cytometry with GFP and 7-AAD. Green dots indicate GFP-positive live cells (figures in the plot indicate GFP%).

B. The comparison of electroporation with GFP protein into Lin$^-$ cells by NEPA21 Super Electroporator. The delivery of GFP protein alone (green dots) versus GFP protein with HITI-cDNA ex2-8 plasmid (black dots) was analyzed by flow cytometry. The addition of cDNA significantly reduces the efficiency of protein delivery. Mann-Whitney one-tailed U test: $*p < 0.05$.

C. Assessment of off-target InDels. Putative off-target sites for Cas9/RNP-il2rg^{in1} were analyzed by Surveyor assay in RNP+HITI-cDNA ex2-8 treated Lin$^-$ cells. No specific mutations were observed. Off, putative off-targets; On-target, *Il2rg* Intron 1; NC, negative control.

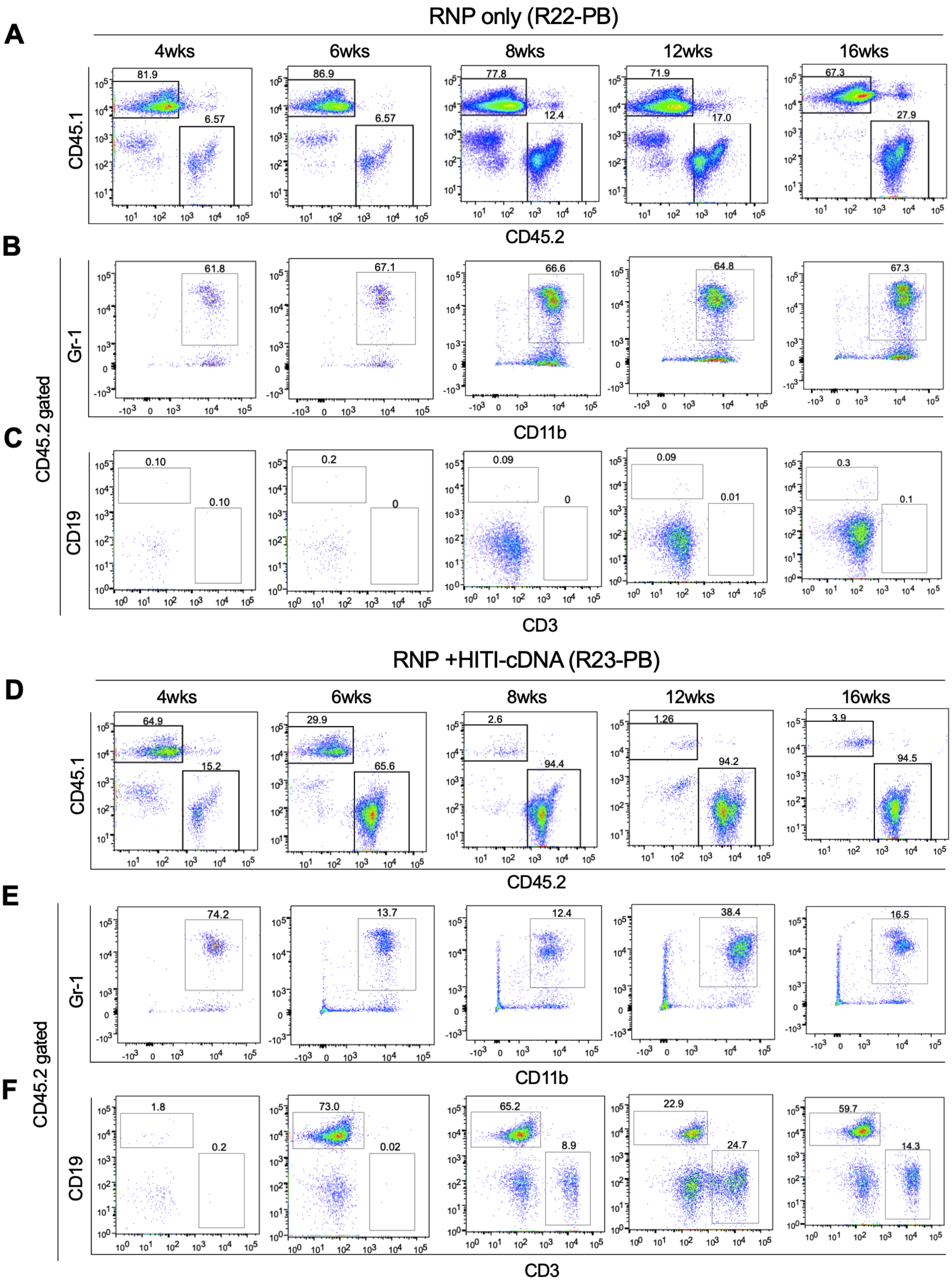

Figure S6. Time course study of donor-derived cells in the primary recipients.

A-C. Plots of CD45.1 (host) and CD45.2 (donor) (**A**), CD45.2-gated Gr-1+ and Cd11b+ granulocytes (**B**), and CD45.2-gated CD19+ B cells and CD3+ T cells (**C**) of a recipient with Lin- cells treated with RNP only.

D-E. Plots of CD45.1/CD45.2 (**D**), CD45.2-gated Gr-1+ and Cd11b+ granulocytes (**E**), and CD45.2-gated CD19+ B cells and CD3+ T cells (**F**) of a recipient with Lin- cells treated with RNP+HITI-cDNA.

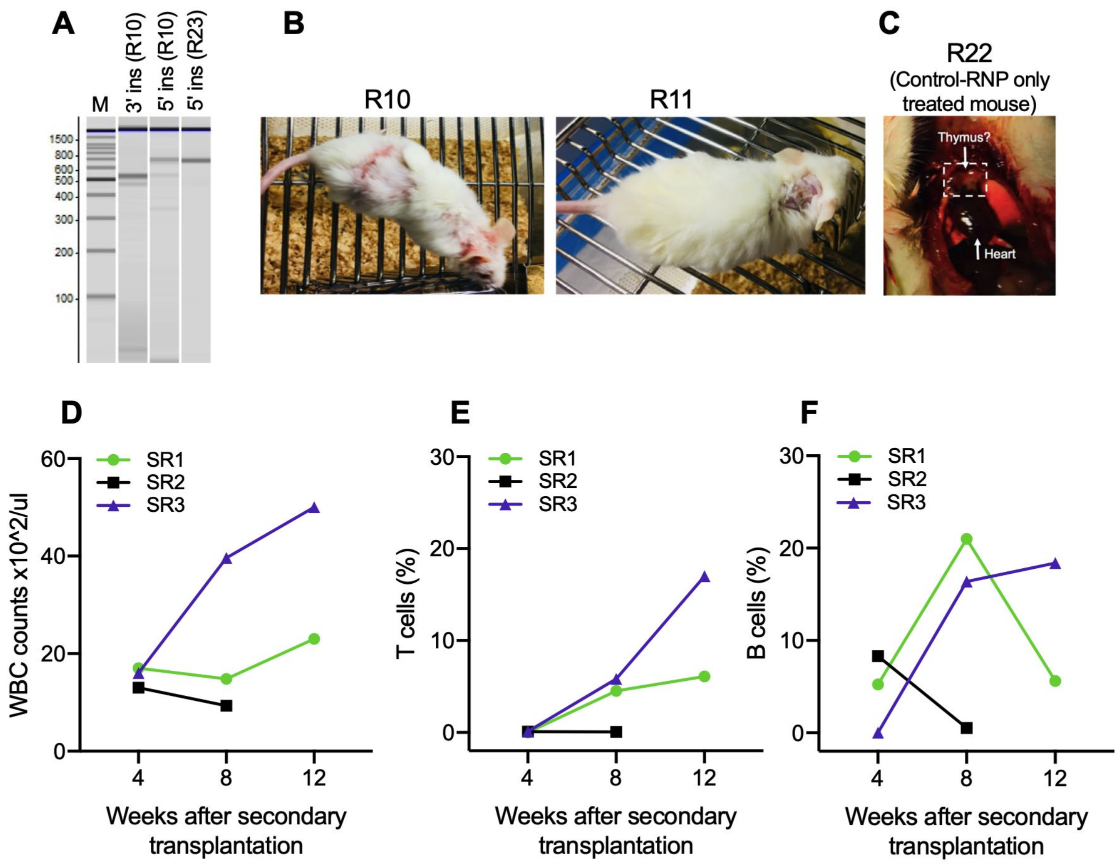

Figure S7. Phenotypical correction of *Il2rg*-mutated mice after transplantation of genome-edited HSCs. In primary transplantation, Lin$^-$ cells of *Il2rg*-mutated mice (CD45.2) were transplanted to NOG mice (CD45.1).

A. Genomic PCR confirming the integration of HITI-cDNAex2-8 into intron 1 in primary recipients. The 3'-end insertion was confirmed in primary recipients R10 and R23, however, 5'-end insertion was confirmed only in R10.

B. In the transplantation of Lin$^-$ cells of *Il2rg*-mutated mice (CD45.2) to NOG mice (CD45.1) which was allogeneic, graft-versus-host disease (GVHD)-like symptoms such as skin thickening, damage, and rashes appeared after 12 weeks post-transplantation in the primary recipients R10 and R11, suggesting that the reconstituted T lymphocytes were functional.

C. In the control mice (*e.g.* R22) that had received Lin- cells electroporated with the Cas9/RNP-il2rg^{in1}, the development of thymuses was not observed at 20 weeks after transplantation.

D – F. Secondary recipients (SR1-3) of *Il2rg*-mutated mice were transplanted with Lin$^-$ cells from the cured mice after primary transplantation. White blood cell counts **(D)**, and fractions of CD3$^+$ T cells **(E)** and CD19$^+$ B cells **(F)** in the secondary recipients at 4 to 12 weeks after transplantation.

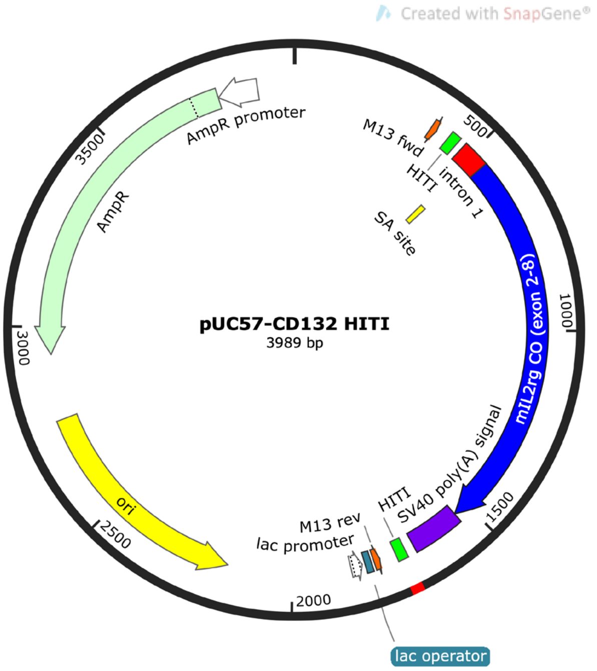

Figure S8. Plasmid map of HITI-cDNAex2-8 (pUC57-CD132-HITI). A plasmid of 3989 base pairs designed for cDNA of *Il2rg* exon 2 to 8. *mIl2rg* CO (exon2-8), codon optimized cDNA of exon 2 to 8 sequence; HITI, Homology independent targeted integration site; AmpR, ampicillin resistance; ori, origin of replication; and SV40 poly A, polyadenylation signal.

M13 forward
GTAAAACGACGGCCAGTGAATTCGAGCTCGGTACCTCGCGAATGCATCTAG
HITI
ACTCCCTCTGGTCCTAGGTTTCCTACCCTAGATAGATCTGTCGA
cDNA_intron1
CCACTGCTGCTTCTTCTGACCAAGAATTCTTTTTCTTTCACTCCACTATTTCATTTTCTTCCCAAACTTAG
cDNA_exon2-8
ATCTGATCCTGACCAGCACCGCGCCGGAACACCTGAGCGCGCCGACCCTGCCGCTGCCGGAGGTTCAATGCTT
CGTTTTCAACATCGAGTATATGAACTGCACCTGGAACAGCAGCAGCGAGCCGCAGGCGACCAACCTGACCCT
GCACTACCGTTATAAGGTGAGCGACAACAACACCTTCCAAGAGTGCAGCCACTACCTGTTTAGCAAGGAAATC
ACCAGCGGTTGCCAGATCCAAAAAGAAGACATTCAGCTGTATCAAACCTTCGTGGTTCAGCTGCAAGATCCGC
AGAAGCCGCAACGTCGTGCGGTGCAGAAACTGAACCTGCAAAACCTGGTTATTCCGCGTGCGCCGGAGAACC
TGACCCTGAGCAACCTGAGCGAGAGCCAGCTGGAACTGCGTTGGAAGAGCCGTCACATCAAAGAGCGTTGCC
TGCAGTACCTGGTGCAATATCGTAGCAACCGTGACCGTAGCTGGACCGAGCTGATTGTTAACCACGAACCGCG
TTTCAGCCTGCCGAGCGTGGATGAGCTGAAGCGTTACACCTTTCGTGTTCGTAGCCGTTATAACCCGATCTGCG
GTAGCAGCCAGCAATGGAGCAAATGGAGCCAGCCGGTGCACTGGGGCAGCCACACCGTTGAGGAAAACCCG
AGCCTGTTCGCGCTGGAGGCGGTGCTGATCCCGGTTGGTACCATGGGCCTGATCATTACCCTGATTTTCGTGTA
CTGCTGGCTGGAACGTATGCCGCCGATCCCGCCGATTAAGAACCTGGAGGACCTGGTGACCGAATATCAGGG
CAACTTCAGCGCGTGGAGCGGTGTTAGCAAAGGCCTGACCGAGAGCCTGCAACCGGATTACAGCGAGCGTTT
TTGCCACGTTAGCGAAATTCCGCCGAAAGGTGGCGCGCTGGGTGAAGGTCCGGGTGGTAGCCCGTGCAGCCT
GCATAGCCCGTACTGGCCGCCGCCGTGCTACAGCCTGAAGCCGGAAGCGTAA
cDNA_polyA
AACTTGTTTATTGCAGCTTATAATGGTTACAAATAAAGCAATAGCATCACAAATTTCACAAATAAAGCATTTTT
TTCACTGCATTCTAGTTGTGGTTTGTCCAAACTCATCAATGTATCTTATCATGTCTGGATCCTCGAGCGGCCGC
ATCGGATCCCG
HITI
ACTCCCTCTGGTCCTAGGTTTCCTACCCGTCGACTGCAGAGGCCTGCATGCAAGCTTGGCGTAATCATG
M13 reverse
GTCATAGCTGTTTCCTG

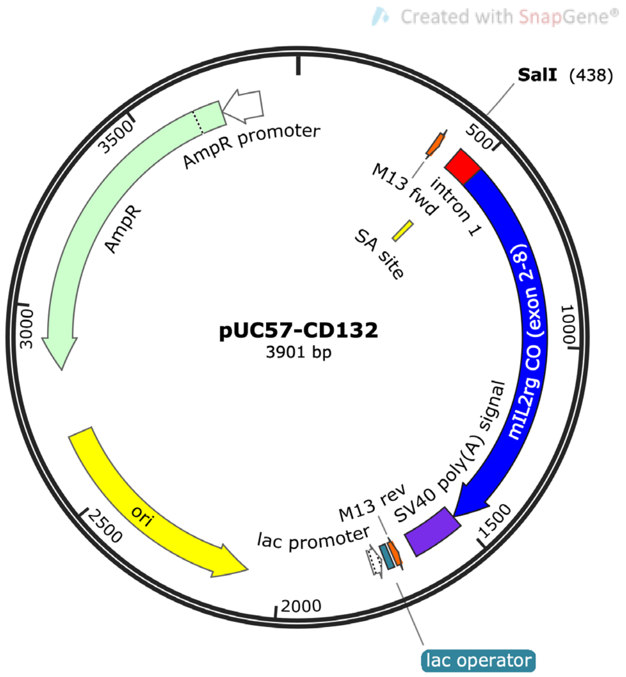

Figure S9. Plasmid map RE-cDNAex2-8 (pUC57-CD132). A plasmid of 3901 base pairs designed for cDNA of *Il2rg* exon 2 to 8. *mIl2rg* CO (exon2-8), codon optimized cDNA of exon 2 to 8 sequence; AmpR, ampicillin resistance; ori, origin of replication; SV40 poly A, polyadenylation signal; and SalI, restriction enzyme site was used for linearization.

M13 forward
GTAAAACGACGGCCAGT GAATTCGAGCTCGGTACCTCGCGAATGCATCTAGATAGATCTGTCGA
SalI cDNA_intron1
GTCGACCACTGCTGCTTCTTCTGACCAAGAATTCTTTTTCTTTCACTCCACTATTTCATTTTCTTCCC
AAACTTAG
cDNA_exon2-8
ATCTGATCCTGACCAGCACCGCGCCGGAACACCTGAGCGCGCCGACCCTGCCGCTGCCGGAGGTT
CAATGCTTCGTTTTCAACATCGAGTATATGAACTGCACCTGGAACAGCAGCAGCGAGCCGCAGGC
GACCAACCTGACCCTGCACTACCGTTATAAGGTGAGCGACAACAACACCTTCCAAGAGTGCAGCC
ACTACCTGTTTAGCAAGGAAATCACCAGCGGTTGCCAGATCCAAAAAGAAGACATTCAGCTGTAT
CAAACCTTCGTGGTTCAGCTGCAAGATCCGCAGAAGCCGCAACGTCGTGCGGTGCAGAAACTGAA
CCTGCAAAACCTGGTTATTCCGCGTGCGCCGGAGAACCTGACCCTGAGCAACCTGAGCGAGAGCC
AGCTGGAACTGCGTTGGAAGAGCCGTCACATCAAAGAGCGTTGCCTGCAGTACCTGGTGCAATAT
CGTAGCAACCGTGACCGTAGCTGGACCGAGCTGATTGTTAACCACGAACCGCGTTTCAGCCTGCCG
AGCGTGGATGAGCTGAAGCGTTACACCTTTCGTGTTCGTAGCCGTTATAACCCGATCTGCGGTAGC
AGCCAGCAATGGAGCAAATGGAGCCAGCCGGTGCACTGGGGCAGCCACACCGTTGAGGAAAACC
CGAGCCTGTTCGCGCTGGAGGCGGTGCTGATCCCGGTTGGTACCATGGGCCTGATCATTACCCTGA
TTTTCGTGTACTGCTGGCTGGAACGTATGCCGCCGATCCCGCCGATTAAGAACCTGGAGGACCTGG
TGACCGAATATCAGGGCAACTTCAGCGCGTGGAGCGGTGTTAGCAAAGGCCTGACCGAGAGCCTG
CAACCGGATTACAGCGAGCGTTTTTGCCACGTTAGCGAAATTCCGCCGAAAGGTGGCGCGCTGGG
TGAAGGTCCGGGTGGTAGCCCGTGCAGCCTGCATAGCCCGTACTGGCCGCCGCCGTGCTACAGCCT
GAAGCCGGAAGCGTAA
cDNA_polyA
AACTTGTTTATTGCAGCTTATAATGGTTACAAATAAAGCAATAGCATCACAAATTTCACAAATAAA
GCATTTTTTTCACTGCATTCTAGTTGTGGTTTGTCCAAACTCATCAATGTATCTTATCATGTCTGGAT
CCTCGAGCGGCCGCATCGGATCCCTGGCGTAATCATG
M13 reverse
GTCATAGCTGTTTCCTG

Supplemental Tables S1-S5 can be downloaded from a separate Excel file

Supplementary Table S6. gRNA list

**Name**	**gRNA sequence**
m*Il2rg* Intron 1 SpCas9 gRNA 1	GTAGGAAACCTAGGACCAGA-GGG
m*Itga2b* exon 1 SpCas9 gRNA 1	GCACTAGGACCCAAGAACAG-TGG
m*Itga2b* exon 1 SpCas9 gRNA 2	ACTTGCGTTTAGGGCCCTGG-GGG

Supplementary Table 7. Primer list

	Primer	Direction	Sequence	Purpose
1	m*Itgα2b* primer 1F	Forward	GGTGAGCTTTCCTGGAGAGGAAG	Detection of mutation by Surveyor assay at *Itgα2b* site
2	m*Itgα2b* primer 1R	Reverse	TCCACTCAGACCGGAGAACTGAC	Detection of mutation by Surveyor assay at *Itgα2b* site
3	m*Itgα2b* primer 2F	Forward	GACAGGCAGACATTTTGTCTGGT	Detection of mutation by Surveyor assay at *Itgα2b* site
4	m*Itgα2b* primer 2R	Reverse	CTAGAGCAGACCCACAGGAGAG	Detection of mutation by Surveyor assay at *Itgα2b* site
5	m*Il2rg* Intron 1_1F	Forward	GAAAAGGTGGCTGGGAATGATGGT	Detection of mutation by Surveyor assay at *Il2rg* site
6	m*Il2rg* Intron 1_1R	Reverse	AGCTAGCCTCATCTGGTCTGAACT	Detection of mutation by Surveyor assay at *Il2rg* site
7	m*Il2rg*_exon2-4_DS_primer_1F_barcode	Forward	CGCTGATCAAAGGAAATGTATGGGTGGGGAGG	For amplicon sequence to multiplex samples
8	m*Il2rg*_exon2-4_DS_primer_2F_barcode	Forward	AACGTGATAAAGGAAATGTATGGGTGGGGAGG	For amplicon sequence to multiplex samples
9	m*Il2rg*_exon2-4_DS_primer_3F_barcode	Forward	AAACATCGAAAGGAAATGTATGGGTGGGGAGG	For amplicon sequence to multiplex samples
10	m*Il2rg*_exon2-4_DS_primer_4F_barcode	Forward	ATGCCTAAAAAGGAAATGTATGGGTGGGGAGG	For amplicon sequence to multiplex samples
11	m*Il2rg*_exon2-4_DS_primer_1R_barcode	Reverse	ACCACTGTTGTTCAGGGGCTGTAGAAGTCAG	For amplicon sequence to multiplex samples
12	m*Il2rg*_exon2-4_DS_primer_2R_barcode	Reverse	AGTGGTCATGTTCAGGGGCTGTAGAAGTCAG	For amplicon sequence to multiplex samples
13	NHEJ insertion check 1F	Forward	CTGGTGACCGAATATCAGGGCA	To confirm the integration of partial-cDNA at 3'-end
14	NHEJ insertion check 1R	Reverse	CCCTCTGCACACTTCGTTTTAGTCA	To confirm the integration of partial-cDNA at 3'-end
15	NHEJ insertion check 2F	Forward	TCCACCGGAAGCTACGACAAAAG	To confirm the integration of partial-cDNA at 5'-end
16	NHEJ insertion check 2R	Reverse	TGAACCACGAAGGTTTGATACAGCTG	To confirm the integration of partial-cDNA at 5'-end
17	HITI sequence primer 1F	Forward	GCAGATTGTACTGAGAGTGCACCA	To check pUC57-CD132-HITI plasmid sequence
18	HITI sequence primer 1R	Reverse	TCATTAATGCAGCTGGCACGACA	To check pUC57-CD132-HITI plasmid sequence
19	HITI primer 1F	Forward	CTAGGTTTCCTACCCTAGATAGATCTGTCG	To construct HITI sequence at 5'-end of pUC57-CD132 plasmid
20	HITI primer 1R	Reverse	GACCAGAGGGAGTCTAGATGCATTCGCGAG	To construct HITI sequence at 5'-end of pUC57-CD132 plasmid
21	HITI primer 2F	Forward	TAGGTTTCCTACCCGTCGACTGCAGAGGCC	To construct HITI sequence at 3'-end of pUC57-CD132 plasmid
22	HITI primer 2R	Reverse	GGACCAGAGGGAGTCGGGATCCGATGCGGC	To construct HITI sequence at 3'-end of pUC57-CD132 plasmid
